# Neurodevelopmental Outcomes of Extremely Preterm Infants Randomized to Stress Dose Hydrocortisone

**DOI:** 10.1371/journal.pone.0137051

**Published:** 2015-09-16

**Authors:** Nehal A. Parikh, Kathleen A. Kennedy, Robert E. Lasky, Jon E. Tyson

**Affiliations:** 1 Department of Pediatrics, University of Texas Medical School at Houston, Houston, TX, United States of America; 2 Center for Perinatal Research, The Research Institute at Nationwide Children’s Hospital, Columbus, OH, United States of America; 3 Department of Pediatrics, The Ohio State University College of Medicine, Columbus, OH, United States of America; 4 Division of Neonatology, Nationwide Children’s Hospital, Columbus, OH, United States of America; 5 Center for Clinical Research and Evidence-Based Medicine, University of Texas Medical School at Houston, Houston, TX, United States of America; Emory University School of Medicine, UNITED STATES

## Abstract

**Objective:**

To compare the effects of stress dose hydrocortisone therapy with placebo on survival without neurodevelopmental impairments in high-risk preterm infants.

**Study Design:**

We recruited 64 extremely low birth weight (birth weight ≤1000g) infants between the ages of 10 and 21 postnatal days who were ventilator-dependent and at high-risk for bronchopulmonary dysplasia. Infants were randomized to a tapering 7-day course of stress dose hydrocortisone or saline placebo. The primary outcome at follow-up was a composite of death, cognitive or language delay, cerebral palsy, severe hearing loss, or bilateral blindness at a corrected age of 18–22 months. Secondary outcomes included continued use of respiratory therapies and somatic growth.

**Results:**

Fifty-seven infants had adequate data for the primary outcome. Of the 28 infants randomized to hydrocortisone, 19 (68%) died or survived with impairment compared with 22 of the 29 infants (76%) assigned to placebo (relative risk: 0.83; 95% CI, 0.61 to 1.14). The rates of death for those in the hydrocortisone and placebo groups were 31% and 41%, respectively (*P* = 0.42). Randomization to hydrocortisone also did not significantly affect the frequency of supplemental oxygen use, positive airway pressure support, or need for respiratory medications.

**Conclusions:**

In high-risk extremely low birth weight infants, stress dose hydrocortisone therapy after 10 days of age had no statistically significant effect on the incidence of death or neurodevelopmental impairment at 18–22 months. These results may inform the design and conduct of future clinical trials.

**Trial Registration:**

ClinicalTrials.gov NCT00167544

## Introduction

Despite advances in perinatal care, one out of every two extremely low birth weight (ELBW; BW ≤1000g) infants develop bronchopulmonary dysplasia (BPD) and/or neurodevelopmental impairments [1], [2]. Bronchopulmonary dysplasia is characterized by systemic inflammation, which may represent one of the potential links between BPD, abnormal brain development, and neurodevelopmental impairments [3], [4]. Physiologic doses of hydrocortisone prescribed in the first week of life do not appear to affect survival without BPD or survival without neurodevelopmental impairments in ELBW infants [5], [6]. However, important subgroup differences may be present and deserve further study [5]. Stress doses of hydrocortisone are increasingly prescribed in the neonatal intensive care unit for prevention or treatment of BPD and for hypotension presumed to be secondary to relative adrenal insufficiency [7], [8], [9]. Hydrocortisone is one of the 15 most frequently prescribed medications in ELBW infants given neonatal intensive care [8]. Despite their widespread use, the effects of stress doses of hydrocortisone or of dosing after a week of age on neurodevelopmental outcomes have not been assessed in randomized trials. It is unknown if these drugs reduce neurodevelopmental impairments [5] by reducing risk factors such as systemic inflammation or mechanical ventilation [10], [11] or perhaps increase impairment risk as recently reported in an observational study that prescribed high cumulative doses of hydrocortisone [12], potentially through mechanisms similar to dexamethasone therapy [13], [14], [15], [16].

We conducted this pilot randomized, placebo-controlled trial of hydrocortisone to study the short- and long-term safety and efficacy of stress doses of hydrocortisone administered after a week of age to ventilator-dependent ELBW infants for prevention of BPD and neurodevelopmental impairments. Corticosteroid therapy and BPD affect neonatal regional and total brain volumes [13], [17] and brain volumes may be predictive of neurodevelopmental impairments [18], [19], [20]. We previously reported no significant differences in brain volumes or respiratory outcomes in ELBW infants randomized to hydrocortisone versus placebo before the first discharge home [21]. The goal of this follow-up study was to determine whether hydrocortisone therapy prescribed at stress doses after 10 days of age alters the incidence of survival without neurodevelopmental impairments at a corrected age of 18 to 22 months.

## Methods

This randomized parallel group double-blind trial was conducted at and approved by the joint Institutional Review Board of the University of Texas Houston Medical School and Children’s Memorial Hermann Hospital. Written informed consent was obtained from a parent or guardian of every study infant.

### Participants

Extremely low birth weight infants cared for at Children’s Memorial Hermann Hospital between the ages of 10 and 21 days were eligible for inclusion if they were ventilator-dependent and their respiratory index score (mean airway pressure X fraction of inspired oxygen) [22] was ≥2.0 and stable or increasing or if the respiratory index score was ≥3.0 when improvement was noted in the previous 24-hour period. The exclusion criteria and data and safety monitoring plan have been reported previously [21].

### Interventions

We enrolled 64 infants between November 2005 and September 2008 and randomly assigned them to a tapering 7-day course of hydrocortisone sodium succinate (Solu-Cortef, Pfizer, New York, NY) every 12 hours (3 mg/kg per day for first 4 days, 2 mg/kg per day for 2 days and 1 mg/kg per day for 1 day; total of 17 mg/kg over 7 days) or an equivalent volume of identical appearing 0.9% sterile saline placebo. The IV route was preferred. Randomization was stratified by birth weight (≤750g or 751–1000g) and respiratory index score (2.0–4.0 or >4.0), using a blocked random allocation (1:1) with variable block sizes. Infants with a respiratory index score between 2.0 and 4.0 would typically be on conventional mechanical ventilation with peak inspiratory pressures ranging from 20–30 cm H2O, end expiratory pressures of 5–6, and inspired oxygen fraction between 0.30–0.40. Such infants were at greater than 75% risk of BPD or death based on local data and results from a multicenter trial [22]. An individual not involved with the study generated the random allocation sequence. Access to this sequence assignment was limited to two study pharmacists. The allocation remained unknown to the members of the clinical and research teams. Details about interim analysis, independent data monitoring, co-interventions, guidelines for clinical use of postnatal corticosteroids, sample size determination, and short-term outcomes have been reported previously [21]. Follow-up assessments were performed between October 2007 and November 2010.

### Neurodevelopmental Impairments

We examined death before 18 months corrected age or survival with one or more of the following neurodevelopmental impairments assessed at 18 to 22 months corrected age: cognitive delay, language delay, cerebral palsy (CP), hearing loss requiring amplification, and bilateral blindness. We defined cognitive delay as a Cognitive Score less than 80 and language delay was defined as a Language Score less than 80 on the Bayley Scales of Infant and Toddler Development, Third Edition [23]. Children who could not be tested due to severe developmental delay were assigned a Cognitive Score of 54 and a Language Score of 46, as specified in the Bayley III instruction manual. A certified examiner blinded to group assignment performed all Bayley testing [24]. Bilateral deafness was defined as bilateral hearing loss requiring amplification and bilateral blindness as bilateral vision loss with only form or shadow vision or no useful vision. All children underwent a complete neurologic physical examination to assess gross motor function and ascertain the presence of CP by certified examiners who were trained annually [24], [25]. Cerebral palsy was defined as the presence of any two of the following three abnormalities: 1) delay in motor milestones, 2) abnormalities observed in the neuromotor exam (tone, deep tendon reflexes, coordination, and movement), and 3) aberrations in primitive reflexes or postural reactions.

### Secondary Outcomes

We used the Gross Motor Function Classification System to determine the level of gross motor function [26]. A normal level of 0 is assigned if the child can walk 10 steps independently and fluently. A possible level 1 is assigned if the child can walk 10 steps but exhibits toe walking or asymmetric walking. Increasing levels up to the highest score of 5 indicate progressively more serious limitations in gross motor function. Weight, height, and head circumference were measured at 18 to 22 months corrected age. To gauge the presence of ongoing severe respiratory disease, we recorded the use of the following at the time of follow-up assessment: oxygen, mechanical ventilation or continuous positive airway pressure, diuretics, bronchodilators, inhaled steroids, oral or intravenous steroids, and any other medications prescribed for reactive airway disease/asthma (e.g. Singulair).

### Statistical Analyses

We based the sample size calculation on our short-term primary outcome measure of total brain tissue volume at term-equivalent age [21]. All outcome analyses followed the principle of intention-to-treat. The main composite outcome measure at follow-up was neurodevelopmental impairments or death. Analysis of this and all other categorical outcomes was performed using the Mantel-Haenszel test to obtain adjusted relative risks (RR) with 95% confidence intervals. The analyses were stratified for baseline stratification variables (birth weight and respiratory index score). The Mantel-Haenszel test for homogeneity was non-significant. The denominator used to calculate the frequency of each outcome was the number of children for whom status with respect to that outcome was known. Additional categorical outcomes were tested using Fisher’s exact test and continuous outcomes were tested using *t* tests or rank order tests. Two-sided P values of less than .05 were considered to indicate statistical significance. No adjustment for multiple testing was performed. More details about the study protocol can be found in the S1 Protocol.

## Results

**Fig 1:**
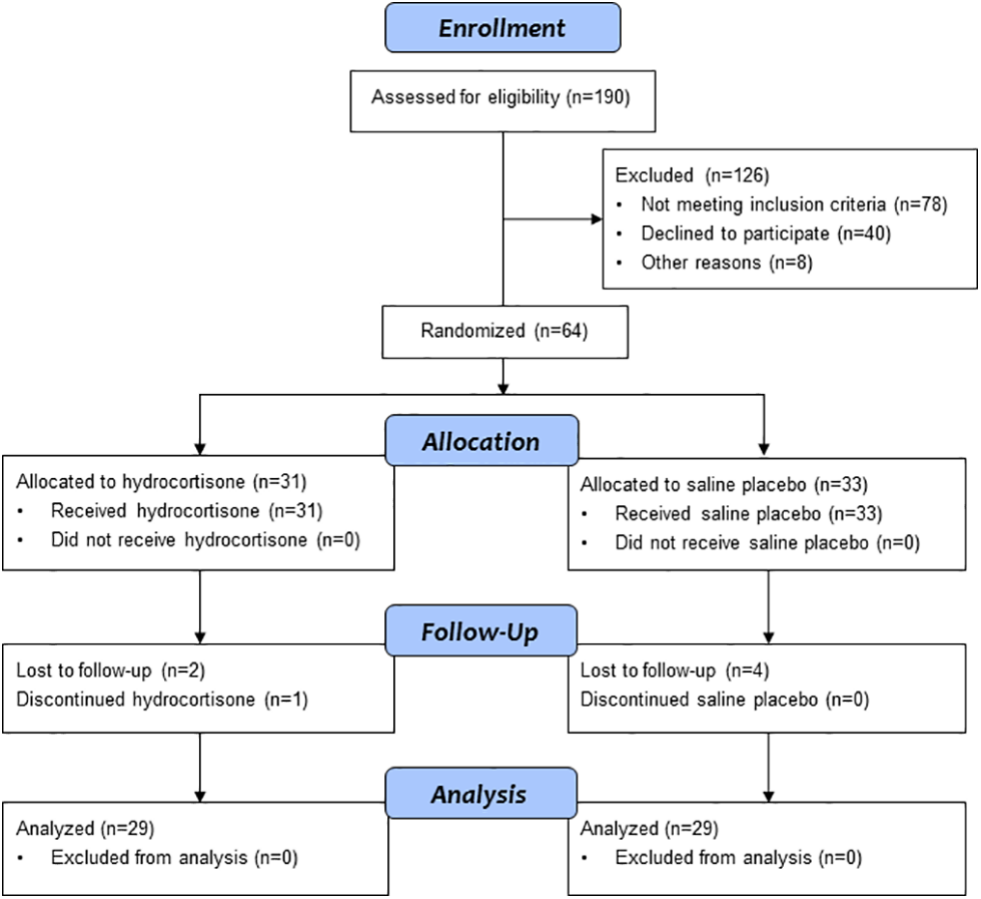
Stress Dose Hydrocortisone Trial Flow Diagram. Flow of study participants from study enrollment to 18 to 22 months corrected age follow-up.

Fig 1 displays the number of infants who were screened for study eligibility, randomized and allocated to hydrocortisone or placebo, lost to follow-up, and the numbers analyzed at 18 to 22 months corrected age. One infant who returned for follow-up could not complete the Bayley exam due to illness. Complete data for an analysis of the main composite outcome of survival without neurodevelopmental impairment were available for 57 (89%) of the infants who were enrolled in the study.

Infant baseline characteristics at birth and at the time of randomization did not differ between infants randomized to hydrocortisone or placebo group (Table 1). The outcomes prior to first hospital discharge were comparable between groups. The children’s corrected ages at follow-up and the characteristics of their maternal caregivers were similar in the two groups.

**Table 1:** Characteristics of the Children and Their Families.

Characteristic			Hydrocortisone Group (N = 28)	Placebo Group (N = 29)
**Infants at baseline** *				
	Birth weight (g), mean±SD		683±107	658±129
	Gestational age (wk), median		25	25
		Interquartile range	24–26	24–26
	Male sex, no. (%)		13 (45)	17 (59)
	Multiple births, no. (%)		6 (21)	8 (28)
	Antenatal steroids (any), no (%)		22 (79)	12 (62)
	Outborn, no. (%)		6 (21)	7 (24)
	Surfactant therapy, no (%)		28 (100)	28 (97)
	Age at randomization (days), median		16	16
		Interquartile range	14–17	13–19
	Respiratory index score at randomization, median		4.1	4.0
		Interquartile range	3.0–5.8	3.4–5.4
**Infants after randomization**				
	Death before first discharge home, no. (%)		9 (31)	10 (35)
	Survival without severe bronchopulmonary dysplasia, no. (%)†		3 (10)	5 (18)
	White matter injury near term-equivalent age, no. (%)§		4 (14)	9 (31)
	Corrected age of surviving children at follow-up (mo), median**		19.4	18.6
		Interquartile range	18.3–21.3	18.2–23.5
**Maternal caregivers at follow-up** ¶				
	Race, White, no. (%)		10 (50)	12 (71)
	Some college or university education, no. (%)		8 (40)	11 (65)
	Household income ≥$40,000, no. (%)€		10 (52)	8 (50)
	Private insurance, no. (%)		4 (20)	6 (35)

* These data are for the 57 children with adequate information for the ascertainment of survival without neurodevelopmental impairment at 2 years corrected age.

† Severe bronchopulmonary dysplasia was defined as oxygen treatment for at least 28 days plus physiologic need for ≥30% oxygen and/or positive pressure at 36 weeks post-menstrual age.

§ White matter injury was defined as the finding of any of the following on cranial ultrasound or brain MRI closest to 36 weeks postmenstrual age: cystic periventricular leukomalacia, porencephalic cyst, parenchymal hemorrhage, and/or ventriculomegaly (with or without intraventricular hemorrhage).

** These data exclude 9 children in the hydrocortisone group and 12 children in the placebo group who died before 18 to 22 months corrected age.

¶ These data exclude mothers of 9 children in the hydrocortisone group and 12 children in the placebo group who died before 18 to 22 months corrected age. Race was self-reported.

€ Data unavailable for one hydrocortisone and one placebo-treated infant.

Hydrocortisone did not improve the likelihood of survival without neurodevelopmental impairment at 18 to 22 months corrected age (Table 2). Nineteen of the 28 infants (68%) randomized to hydrocortisone died or survived with neurodevelopmental impairment, as compared to 22 of the 29 infants (76%) assigned to placebo (RR: 0.83; 95% confidence interval [CI], 0.61 to 1.14). The incidence of death was 31% for those in the hydrocortisone group and 41% for those in the placebo group (*P* = 0.42). The incidence of cognitive delay was 21% (4/19) in infants treated with hydrocortisone and 47% (8/17) in those assigned to placebo, but this was not significantly different between the two groups (RR: 0.46; 95% CI, 0.18 to 1.17). Only one study infant developed severe neurosensory impairment–a placebo treated infant who developed hearing loss requiring amplification. There were no cases of bilateral blindness in either study group. Cerebral palsy was diagnosed in 3 of the 20 survivors (15%) randomized to hydrocortisone and 1 of the 17 infants (6%) assigned to placebo but this difference again was not statistically significant. Three of the four cases of CP were mild and one was severe (hydrocortisone group) (Table 2).

**Table 2:** Primary Outcome of Death or Neurodevelopmental Impairment at Corrected Age of 18 to 22 Months.

Outcome	Hydrocortisone Group	Placebo Group	Relative Risk (95% CI)	P Value
	no./total no. (%)			
Composite				
Death or impairment	19/28 (68)	22/29 (76)	0.83 (0.61–1.14)	0.55
Components				
Death before 18 mo	9/29 (31)	12/29 (41)	0.80 (0.38–1.68)	0.42
Cognitive delay†	4/19 (21)	8/17 (47)	0.46 (0.18–1.17)	0.15
Language delay¶	9/18 (50)	10/17 (59)	0.81 (0.47–1.38)	0.67
Cerebral palsy	3/20 (15)	1/17 (6)	2.26 (0.28–18.3)	0.42

† Cognitive delay was defined as a Bayley Cognitive Score of less than 80.

¶ Language delay was defined as a Bayley Language Score of less than 80.

The rates of gross motor function level of 1 or higher were 25% in the hydrocortisone group and 29% in the placebo group (Table 3). Both study groups had similar mean cognitive and language scores at 18 to 22 months corrected age. At time of follow-up, randomization to hydrocortisone also did not significantly affect the frequency of supplemental oxygen use, positive airway pressure support, or need for respiratory medications for BPD or reactive airway disease. These outcomes are consistent with the lack of statistical differences we previously reported at 36 weeks postmenstrual age in survival without severe BPD, days on supplemental oxygen, and days on positive pressure support. The mean weight, height, and head circumference percentiles were also similar in the two groups at 18 to 22 months corrected age (Table 3) [27].

**Table 3:** Other Outcomes at 18 to 22 Months Corrected Age.*

Outcome	Hydrocortisone Group	Placebo Group	Mean Difference (95% CI)	P Value
Cognitive Score	86.7 (77.7–95.7)	83.9 (71.3–96.5)	2.8 (-11.9, 17.5)	0.70
Language Score	80.6 (69.9–91.2)	77.5 (66.0–89.0)	3.0 (-12.0, 18.1)	0.68
Weight percentile	34.1 (20.0–48.3)	30.7 (18.3–43.1)	3.4 (-14.6, 21.6)	0.70
Height percentile	25.9 (12.6–39.1)	31.8 (17.8–45.7)	-5.9 (-24.4, 12.6)	0.52
Head circumference percentile	41.3 (24.5–58.0)	35.2 (17.3–53.2)	6.0 (-17.6, 29.7)	0.61
	no./total no. (%)		**Relative Risk**	
GMFCS level of 1 to 5†	5/20 (25)	5/17 (29)	0.85 (0.30, 2.45)	1.00
Diuretic use at follow-up	4/20 (20)	2 /17 (12)	1.70 (0.35, 8.17)	0.67
Other respiratory medication use at follow-up¶	8/20 (40)	5/17 (29)	1.36 (0.55, 3.38)	0.73
Supplemental oxygen use at follow-up€	3/20 (15)	2/17 (12)	1.28 (0.24, 6.76)	1.00
Positive airway pressure support at follow-up§	3/20 (15)	2/17 (12)	1.28 (0.24, 6.76)	1.00

* All values are mean (95% CI) unless noted otherwise; reported for survivors with follow-up only.

† GMFCS denotes Gross Motor Function Classification System as defined by Palisano et al [26].

¶ This outcome included use of one or more of the following medications at the time of follow-up assessment: bronchodilators, inhaled steroids, oral/intravenous steroids, and any medications prescribed for reactive airway disease.

€ This outcome was defined as use of supplemental oxygen at the time of follow-up assessment.

§ This outcome was defined as use of mechanical ventilation or nasal continuous positive airway pressure at the time of follow-up assessment.

## Discussion

We conducted this pilot randomized trial to evaluate the neurologic and pulmonary effects of stress dose hydrocortisone in preterm infants. A cumulative dose of 17 mg/kg of hydrocortisone after a week of age demonstrated a clinically meaningful but statistically insignificant reduction in the occurrence of death or impairment, especially cognitive delay, at 18–22 months. Our trial was powered to examine a 2-week delay in brain growth but was underpowered to detect the observed 17% reduction in death or neurodevelopmental impairment. Nevertheless, this is the first trial to report neurodevelopmental outcomes following stress dose hydrocortisone and the relative risk of less than 1 provides some reassurance about the safety of this commonly used medication in ELBW neonates. Prior observational studies reported either no difference [28], [29] or impairments in language and motor skills following neonatal stress dose hydrocortisone therapy [12]. Furthermore, our results can be combined with similar clinical trials in future meta-analyses. Our study hypothesis of improved brain tissue volume and improved survival without impairment was based on a meta-regression of all dexamethasone trials that reported reduced risk of death or CP following dexamethasone administration to infants at >70% baseline risk of death or BPD [10]. The baseline risk of death or severe BPD was 84% in our trial. Although we were unable to confirm our hypothesis, the direction of treatment effect favored hydrocortisone administration. Additional trials and meta-analysis are needed to fully validate our hypothesis.

Lower physiologic doses of hydrocortisone have been studied in eight randomized trials, all initiated before a week of age for prevention/treatment of BPD. A meta-analysis of these trials (totaling 880 infants) concluded that early life treatment with physiologic doses of hydrocortisone “has few beneficial or harmful effects and cannot be recommended for prevention of BPD” [6]. Yet, hydrocortisone remains one of the most frequently prescribed medications in ELBW infants [8] and little is known about the potential beneficial or harmful effects of the commonly used higher stress doses of hydrocortisone. Only one prior study has reported on the pulmonary effects of stress dose hydrocortisone [30]; this non-randomized cohort study found an acute reduction in oxygen dependency in 25 hydrocortisone-treated preterm infants as compared to 25 untreated infants matched for birth weight, gestational age, and degree of respiratory distress syndrome. However, no respiratory outcomes were reported beyond 14 days from steroid initiation and rates of BPD may have been unaffected. In our trial, ELBW infants randomized to hydrocortisone and placebo exhibited comparable incidence of survival without BPD at 36 weeks postmenstrual age and pulmonary outcomes at 18 to 22 months. Lack of benefit could also be explained by our use of a cumulative stress dose of hydrocortisone that was approximately one-fourth the dose used in this observational study.

We limited the total dose of hydrocortisone to relatively lower stress doses to minimize any adverse influence on neurodevelopment. In our pilot trial, we found no adverse effects of hydrocortisone using a cumulative dose of 17 mg/kg on short or long-term development. The van der Heide-Jalving et al. study [30] used a hydrocortisone total dose of 72 mg/kg (equivalent to approximately 3 mg/kg of dexamethasone). This study and subsequent larger cohort studies from the same institution have not reported adverse or beneficial effects of hydrocortisone on MRI findings, IQ, memory testing, or CP [28], [29], [31]. A larger non-randomized study of ELBW infants reported that increasing cumulative exposure to hydrocortisone (maximum total dose and duration: 50 mg/kg and 38 days) was associated with lower receptive and expressive language skills at 8 months corrected age and worse motor skills at 20 months corrected age as compared to untreated ELBW infants [12]. Even though lower doses of hydrocortisone do not appear to benefit the lungs [6], [21] they may improve cognitive outcomes. In secondary analyses of the PROPHET trial [5], ELBW infants randomized to physiologic doses of hydrocortisone exhibited a 53% significant reduction in the odds of developing cognitive delay as compared to placebo treated infants. In our smaller trial, we observed a 54% reduced risk in cognitive delay, though this did not reach statistical significance. Randomized trials sufficiently powered to evaluate neurodevelopment outcomes–similar to the ongoing Hydrocortisone for BPD trial–are ideally positioned to answer this question [32].

The doses we used in this trial are similar to clinically used stress doses of hydrocortisone for hypotension and vasopressor dependence in preterm infants. However, it is unclear if our findings can be extrapolated to this group of infants. Only seven small randomized trials (N = 144) have assessed the safety and benefits of stress dose hydrocortisone in this population and none of them reported neurodevelopmental outcomes [9]. Randomized trials with neurodevelopmental follow-up are needed to determine long-term safety and potential benefits.

Our trial has several limitations. It was not powered to examine survival without neurodevelopmental impairments. While larger trials and/or meta-analysis of several smaller trials are needed to resolve this issue, our trial nevertheless provides initial evidence from a randomized trial of the potential safety of clinically used stress doses of hydrocortisone on neurodevelopmental outcomes. Trial participants experienced higher than expected incidence of mortality, likely as a result of inclusion of high risk infants; this was reflected in the high respiratory index scores at enrollment, large proportion of small for gestational age infants, and frequent occurrence of late onset sepsis in both groups [21]. Nevertheless, mortality was not statistically different between infants randomized to hydrocortisone or placebo. Inclusion criteria for future trials should include lower risk infants. Last, we determined the presence of CP at 18 to 22 months corrected age rather than 24 or 30 months corrected age. Recent evidence suggests that diagnosis of CP is less accurate at 18 months corrected age [33]. Longer follow-up is also needed to accurately assess cognitive outcomes [34].

The American Academy of Pediatric Policy Statement on the use of postnatal corticosteroids for BPD [16] noted the lack of trial data to make a recommendation regarding treatment with higher doses of hydrocortisone. Our results provide initial evidence from a pilot randomized trial of the effects of higher dose hydrocortisone administered to high-risk ELBW infants after one week of postnatal age on neurodevelopmental impairments or death and respiratory outcomes. Additionally, we found preliminary evidence of safety and possible benefit for stress dose hydrocortisone that support the design and conduct of future stress dose hydrocortisone trials.

## Supporting Information

S1 CONSORT Checklist CONSORT Checklist. (DOC)

S1 Protocol Trial protocol. Hydrocortisone Trial Study Protocol.
(PDF)
