# Supplementary material for: Neurodevelopmental Outcomes of Extremely Preterm Infants Randomized to Stress Dose Hydrocortisone
Source: PLoS One. 2015 Sep 16;10(9):e0137051. doi: 10.1371/journal.pone.0137051 (PMC4573756; doi:10.1371/journal.pone.0137051)
Supplement: S1 Protocol — Hydrocortisone Trial Study Protocol. (PDF) [file pone.0137051.s002.pdf]

PROTOCOL TITLE

A Randomized Trial of Hydrocortisone in Very Preterm Infants at High Risk for Neurologic and Pulmonary Impairments

Principal Investigator:

Nehal A. Parikh, D.O.
Division of Neonatal-Perinatal Medicine
University of Texas Medical School at Houston

Co-Investigators:

Jon E. Tyson, MD, MPH, Kathleen A. Kennedy, MD, MPH, Robert E. Lasky, PhD.
Center for Clinical Research and Evidence-Based Medicine
University of Texas Medical School at Houston

Patrick G. Brosnan, M.D.
Head, Division of Pediatric Endocrinology
University of Texas Medical School at Houston

Supported by:

The National Institute of Neurological
Disorders and Stroke (NINDS)
Grant # K23 NS048152

University Clinical Research Center
University of Texas Medical School at Houston
Grant # M01- RR 002558

TABLE OF CONTENTS

		Page
CLINICAL SITES PARTICIPATING IN THE STUDY		4
STUDY TEAM ROSTER		5
PRÉCIS		6
**1.**	**STUDY OBJECTIVES**	7
1.1	Primary Objective	7
1.2	Secondary Objectives	7
**2.**	**BACKGROUND**	7
2.1	Rationale	7
2.2	Supporting Data	10
**3.**	**STUDY DESIGN**	12
**4.**	**SELECTION AND ENROLLMENT OF SUBJECTS**	12
4.1	Inclusion Criteria	13
4.2	Exclusion Criteria	13
4.3	Study Enrollment Procedures	13
**5.**	**STUDY INTERVENTIONS**	14
5.1	Interventions, Administration, and Duration	14
5.2	Concomitant Interventions	14
5.3	Adherence Assessment	15
**6.**	**CLINICAL AND LABORATORY EVALUATIONS**	15
**7.**	**MANAGEMENT OF ADVERSE EXPERIENCES**	16
**8.**	**CRITERIA FOR INTERVENTION DISCONTINUATION**	17
**9.**	**STATISTICAL CONSIDERATIONS**	17
9.1	General Design Issues	17
9.2	Outcomes	18
9.3	Sample Size and Accrual	19
9.4	Data Monitoring	20
9.5	Data Analyses	20
**10.**	**DATA COLLECTION, SITE MONITORING, AND ADVERSE EXPERIENCE REPORTING**	21

Page

10.1 Records to be Kept …………………………………………………………………..… 21
10.2 Role of Data Management ………………………………………………………..… 22
10.3 Adverse Experience Reporting ………………………………………………….. 22

11. HUMAN SUBJECTS …………………………………………………………..…….. 22

11.1 Institutional Review Board (IRB) Review and Informed Consent ………………... 22
11.2 Subject Confidentiality ……………………………………………………...…… 23
11.3 Study Modification/Discontinuation ……………………………………….…..… 23

12. PUBLICATION OF RESEARCH FINDINGS ……………………………...…….. 23

APPENDICES

I. INFORMED CONSENT FORM 24

II. ADVERSE EVENT FORM 29

III. MRI DETAILS 32

IV. DEFINITIONS – NEUROLOGIC ASSESMENTS 33

V. REFERENCES 34

CLINICAL SITES PARTICIPATING IN THE STUDY

Memorial Hermann Children's Hospital
Nehal A. Parikh, DO, FAAP (PI)
6431 Fannin, MSB 3.236B
Houston, TX 77030
713 500 6470
713 500 5794 fax
Nehal.A.Parikh@uth.tmc.edu

STUDY TEAM ROSTER

Kathleen A. Kennedy, MD, MPH
6431 Fannin, MSB 2.106
Houston, TX 77030
713 500 6708
713 500 0519 fax
Kathleen.A.Kennedy@uth.tmc.edu

Jon E. Tyson, MD, MPH
6431 Fannin, MSB 2.106
Houston, TX 77030
713 500 5651
713 500 0519 fax
Jon.E.Tyson@uth.tmc.edu

Robert E. Lasky, PhD
6431 Fannin, MSB 2.104D
Houston, TX 77030
713 500 6708
713 500 0519 fax
Robert.E.Lasky@uth.tmc.edu

Patrick G. Brosnan, MD
6431 Fannin, MSB 3.122
Houston, TX 77030
713 500 5646
Patrick.G.Brosnan@uth.tmc.edu

Georgia E. McDavid, RN
Study Coordinator
6431 Fannin, MSB 3.252
Houston, TX 77030
713 500 5734
713 500 5794 fax
Georgia.E.McDavid@uth.tmc.edu

PRÉCIS

Study Title

A Randomized Trial of Hydrocortisone in Very Preterm Infants at High Risk for Neurologic and Pulmonary Impairments

Objectives

a) Delineate the effects of hydrocortisone on cerebral tissue volumes and bronchopulmonary dysplasia (BPD) in extremely low birth weight (ELBW; BW ≤ 1000g) infants.
b) Evaluate the effects of hydrocortisone and correlate volumetric MRI findings to neurodevelopmental outcomes at 18 to 22 month adjusted age.

Design and Outcomes

This will be a single-center, phase II, double-masked, randomized controlled trial of systemic hydrocortisone or saline placebo administered to ventilator-dependent ELBW infants who are at high risk for developing BPD. As infants with BPD are also at high risk for neurologic injury, reduction of BPD should improve neurologic outcomes as well. We will therefore assess total cerebral volumes as measured by volumetric brain MRI at 38 weeks post-menstrual age (PMA) as our primary outcome. Our principal secondary outcome to be evaluated will be neurosensory impairments at 18 to 22 months adjusted age. Additional outcomes to be evaluated include, regional brain volumes, duration of mechanical ventilation, duration of oxygen (O_2) requirement, and diagnosis of BPD (as assessed by O_2 therapy at 36 weeks PMA plus total O_2 duration ≥ 28 days with classification of severity).

Interventions and Duration

Hydrocortisone group: Hydrocortisone sodium succinate 1.5 mg/kg/dose IV q 12 hours for days 1-4 followed by 1 mg/kg/dose days 5-6 and 0.5 mg/kg/dose on day 7.
Placebo group: Saline placebo with equivalent volumes given IV q 12 hours for 7 days.

After the 7 day intervention period, infants will be followed until completion of brain MRI, usually performed at 38 weeks PMA. Therefore enrolled infants will be considered "in the study" from the age of 10 to 21 days (enrollment period) up to 38 weeks PMA. All ELBW infants at our institution are also followed post-discharge until 18 to 22 months adjusted age for neurosensory impairments. The study protocol will further improve the tracking and follow-up rates for these infants and report neurosensory outcomes for all surviving enrolled infants.

Sample Size and Population

We will need to enroll a total of 56 mechanically ventilated ELBW infants to determine the primary outcome at 38 weeks PMA in a total of 44 infants (allowing for ~20% mortality post-enrollment). Randomization will be blocked and stratified by birth weight [≤ or >750 g] and by severity of lung disease [Respiratory index score (RIS) 2.0 to 4 or > 4].

1 STUDY OBJECTIVES

1.1 Primary Objective

To perform a randomized controlled trial and determine the effects of a 7-day course of hydrocortisone as compared to placebo administered to ELBW infants at high risk for BPD. We hypothesize that the experimental treatment will result in an improved total cerebral tissue volume (total gray and white matter) comparable to 2 weeks in cerebral growth as measured by volumetric MRI at 38 weeks PMA.

Null Hypothesis: Among ELBW infants at high risk for BPD, randomization to seven days of hydrocortisone will result in a comparable total cerebral tissue volume as compared to infants randomized to placebo.

1.2 Secondary Objectives

To evaluate neurosensory outcomes at 18 to 22 months adjusted age of hydrocortisone and placebo treated infants and to correlate various regional and global cerebral volumes measured at discharge with such functional outcomes. Should steroid therapy result in measurable benefits in cerebral tissue volume and/or functional outcome(s), design and execution of a larger multicenter randomized trial would be an important objective of this proposed pilot study.

2 BACKGROUND

2.1 Rationale

Neurosensory impairments remain prevalent in the ELBW population affecting close to 50% of survivors.[1,2] Bronchopulmonary dysplasia, a chronic inflammatory lung disease of prematurity, is also common and develops in approximately 30% to 40% of all ELBW infants with a higher predilection for those with lower birth weights.[3] Extremely low birth weight infants with BPD are at a greater risk of neurosensory impairments as compared to comparable infants without BPD. Several cohort studies have clearly demonstrated significant neurologic abnormalities, including poor head growth, progressive neurologic deterioration, and evidence of global neuronal loss (at autopsy) associated with severe BPD.[4,5,6,7,8] Schmidt et al recently demonstrated from a large international cohort that BPD is an independent and important risk factor in ELBW infants for the prediction of long-term neurosensory impairments. This risk was comparable to the risk imposed by severe brain injury as evidenced by cranial ultrasonography.[2] The underlying genesis of neuronal toxicity seen in infants with BPD is likely multifactorial. Chronic intermittent episodes of hypoxia and poor postnatal nutrition and growth have been implicated in the adverse neurodevelopmental outcomes often seen in infants with BPD.[9] Moreover, inflammation beginning with intrauterine infection and further exacerbated by ventilation, oxygen, and postnatal sepsis, may play a direct role in brain injury.[10,11] Prevention or amelioration of BPD would be expected to reduce the incidence of neurosensory impairments in ELBW infants.

Effective prevention of BPD has been elusive with the notable exception of postnatal administration of systemic corticosteroids, dexamethasone in particular. A 30-40% re-

duction in the incidence of the disease is seen when dexamethasone is administered to at risk infants with evolving BPD.[12] While this reduction in BPD should expectedly improve neurologic outcomes, dexamethasone treated infants have unfortunately been shown to have an increased prevalence of cerebral palsy (CP) and other neurodevelopmental impairments,[12,13] presumably because the direct neurotoxic effects of dexamethasone overshadow the indirect beneficial effect that reducing BPD might have. Meta-analyses of all randomized trials of steroids administered before 96 hours of life with neurological follow-up demonstrated a significant increased risk of CP (RR 1.69; 95% CI: 1.20, 2.38).[39] A critical analysis of all randomized trials of dexamethasone in human newborns suggests that neuronal toxicity may have been related to selection of the corticosteroid (i.e. dexamethasone), type of preservatives in the formulation (i.e. sulfites), inappropriately high dose selection, early postnatal use of steroids, and/or poor selection strategy of low risk patients.[14,15,16]

Choice of corticosteroid

The selection of dexamethasone over other synthetic corticosteroids for prevention trials of BPD appears to have been arbitrary.[17] Dexamethasone formulations used in North America and Europe contain sulfites as an additive; dexamethasone with sulfites has been shown to be neurotoxic in animal studies whereas preservative-free dexamethasone and betamethasone had no effect.[18,19] A case-control study of 833 neonates also suggested that antenatal betamethasone, with a structure very similar to dexamethasone, reduced the incidence of cystic periventricular leukomalacia, whereas antenatal dexamethasone with sulfite additives appeared to increase its incidence.[20] Betamethasone has not been tested in preterm infants after birth possibly due to the short supply status of this synthetic corticosteroid. The use of methylprednisolone, a less potent corticosteroid, has been shown to have beneficial effects on pulmonary function with less glucose intolerance and reduced periventricular leukomalacia.[21] Effects on long-term neurodevelopmental status are unknown however.

Watterberg et al conducted a pilot randomized trial of hydrocortisone in physiologic replacement doses for presumed adrenal insufficiency (1 mg/kg/day: equivalent to 0.03 mg/kg/day of dexamethasone) in a small group of ELBW infants. Therapy for 12 days initiated soon after birth resulted in improved respiratory status and reduced risk of BPD defined by an O_2 need at 36 weeks.[22] A larger follow-up trial with planned neurologic assessments to test the effects of this same hydrocortisone regimen was halted prematurely due to an increased risk of intestinal perforations in the steroid arm of the trial.[23] This risk was seen almost exclusively in infants given hydrocortisone while being treated with indomethacin (most within <48 hours of life). A multi-center NICHD trial of dexamethasone was also prematurely halted due to an increased risk of intestinal perforation, again confined to selected patients treated with dexamethasone and indomethacin primarily in the first week of life.[24] The combination of indomethacin and dexamethasone has been shown to deplete endothelial and inducible nitric oxide synthase in the intestinal submucosa in vitro, thereby leading to diminished blood flow and intestinal perforation.[25] This combination of drugs therefore should be avoided. Other adverse effects commonly seen with dexamethasone were not seen with this less potent hydrocortisone formulation. Long-term effects of hydrocortisone need to be properly evaluated before routine use becomes common practice. Preliminary evidence from a case-control study suggests that hydrocortisone at doses of 5mg/kg/day prescribed for 22 days did not result in neurodevelopmental delay evaluated at 5 -7 years as compared to matched controls.[26] Relative adrenal insufficiency in the smallest of premature survivors has been

hypothesized as a potential factor contributing to the development of BPD.[27] If this was a significant contributor to BPD rates, the use of a more physiologic steroid such as hydrocortisone with its mineralocorticoid actions in addition to glucocorticoid effects and lack of sulfite preservatives would be more desirable.

Optimizing dose and duration of therapy

The appropriate dose and duration of dexamethasone selected for BPD prevention trials appears to have been arbitrary as well.[17] Most trials reporting adverse neurodevelopmental outcomes have used dexamethasone at doses of 0.5 mg/kg/day over 7 to 42 day tapering treatment periods. This dose represents 10 to 20 times daily basal cortisol production,[15] and is greater than 10 times the dose needed for maximal inhibition of interleukin-8 release and neutrophil migration in vitro[28]. Such high pharmacologic doses of dexamethasone have been postulated as causes of poor growth, aggravated glucose intolerance, hypertrophic cardiomyopathy, leukocytosis, and possibly diminished neutrophil function.[29,30,31,32,33] In a small non-randomized study of preterm infants by Murphy et al,[34] administration of dexamethasone at starting doses of 0.5 mg/kg/day for up to 42 days resulted in 30% smaller total cerebral volumes as compared to brains of untreated infants. This corresponds to a six-week delay in cerebral growth (using the developmental functions reported by Huppi et al[35]). Specifically, the cerebral volume of a 40-week PMA dexamethasone treated newborn was comparable to that of a 34-week PMA newborn. It is unclear how much of this reduction in cerebral volume was a result of dexamethasone treatment and what percentage may have been a result of significant differences in the two study groups. In a somewhat larger cohort study performed by our group, with stricter attention to patient selection, masking, and controlling for confounders, we found a 10% significant reduction in total cerebral volume as assessed by volumetric MRI in dexamethasone treated infants (median duration 7 days) as compared to untreated ones.[36]

More modest doses (0.10-0.20 mg/kg/day of dexamethasone or equivalent to 3-6 mg/kg/day of hydrocortisone) might be expected to provide adequate anti-inflammatory effects (2-4 times the basal secretion rate) with lower toxicity. Recent evidence suggests that a lower dose regimen (0.20 mg/kg/day dose of dexamethasone), administered for a short duration (7 days), may be equally effective on pulmonary inflammatory disease with a more favorable neurodevelopmental outcome.[37,38] Optimizing the dose and duration of therapy necessary to adequately suppress inflammation while minimizing toxicity remains an important unanswered challenge.

Timing of therapy

The optimal age to initiate postnatal steroid therapy has not been established. As the disease process often begins in the perinatal period with intrauterine infection, use of mechanical ventilation and oxygen and resulting surge of inflammatory cytokines, earlier use of steroids may theoretically attenuate pulmonary inflammatory injury and minimize resulting fibrosis. Indeed, the relative risk of BPD is lower in studies using steroids at 7-14 days as compared to >21 days of age.[12] Early therapy in the first week of life also results in a better risk reduction as compared to later therapy. Cochrane meta-analyses of all early (<96 hours of life)[39], moderately early (7-14 days)[40], and delayed (> 3weeks)[41] corticosteroid treatment for the prevention of BPD however showed an increased risk of CP with early treatment only. Moderately early treatment in higher risk neonates does not appear to increase risk of cerebral palsy (RR 0.83, 95% CI: 0.39, 1.74).[40,42]

In ELBW infants, the incidence of isolated intestinal perforation and patent ductus arteriosus necessitating indomethacin therapy is highest in the first week of life. As opposed to studies of early therapy and risk of intestinal perforation, administration of dexamethasone after 21 days of age does not appear to increase risk of intestinal perforation. Initiation between 7 and 14 days of age has shown no increase in necrotizing enterocolitis (another gastrointestinal disorder that may cause intestinal perforation and that may be related to or confused with spontaneous intestinal perforation); spontaneous intestinal perforation was not reported. In summary, these studies suggest that the gastrointestinal side effects of postnatal steroid treatment can be minimized by delaying treatment until after 7-10 days of age and by avoiding concomitant administration of steroids with indomethacin.

Target patient selection

Treatment of all extremely preterm infants soon after birth with postnatal preventive therapy exposes some infants at relatively low risk of BPD to adverse neurologic effects with minimal potential benefit. Delaying treatment until after the first week of life allows for a more accurate selection of infants at higher risk of developing BPD as predicted by their requirements for respiratory support after the first week of life.[43] In a NICHD Network steroid trial of infants less than 1500g BW with a respiratory index score (RIS; mean airway pressure x fraction of inspired oxygen) ≥ 2.4 at 10 days of age, 66% of these infants developed BPD, defined as the need for supplemental oxygen at 36 weeks PMA.[44] Infants at high risk of developing BPD are more likely to derive direct pulmonary benefit from steroid treatment and may derive indirect neurologic benefit if steroids decrease the ongoing hypoxia and inflammation associated with BPD.

A meta-regression of all dexamethasone trials with neurodevelopmental follow-up strongly suggests that control infants with the lowest risk of developing BPD (usually those treated early) are most likely to developed adverse effects of dexamethasone such as death or CP. Conversely, infants at the highest risk of developing BPD appear to have no increased risk for mortality or CP.[16] In fact, those infants at greater than 65% risk of developing BPD appeared to have a lower risk of death or developing CP.

2.2 Supporting Data

Recent joint recommendations from the American Academy of Pediatrics and Canadian Paediatric Society state that use of systemic dexamethasone and other alternative corticosteroids should be limited to carefully designed randomized trials and outside the context of such trials its use should be limited to “exceptional clinical situations” (i.e., an infant on maximal ventilator and oxygen support).[45] Since BPD remains prevalent in the ELBW population with limited proven therapies available, clinicians continue to use corticosteroids, although at a much reduced frequency and primarily in those infants at highest risk.[17,46] Further research is urgently needed to identify less toxic but equally effective steroid regimens and to identify the infants for whom the benefits of treatment clearly exceed the risks.[11,17,47,48,49,50]

Recent attempts to uncover a regimen that was effective and safe have been largely unsuccessful with three large randomized trials of low dose (0.15 mg/kg/day) dexamethasone[24] and hydrocortisone in doses of 1 mg/kg/day[23] and 2 mg/kg/day[51] all halted

prematurely due to increased risk of gastrointestinal perforations in the steroid arm of the trial. All three trials initiated steroid treatment in the first 48 hours of life and all allowed the concurrent use of indomethacin. The majority of intestinal perforations occurred in the first few days of life in infants receiving steroids and indomethacin. Use of hydrocortisone in a population of ELBW infants not treated with prophylactic indomethacin has not been shown to result in increased risk of intestinal perforations.[52] The combination of indomethacin and dexamethasone has been shown to deplete endothelial and inducible nitric oxide synthase in the intestinal submucosa in vitro, thereby leading to diminished blood flow and intestinal perforation.[53] None of these trials demonstrated a reduction in rates of BPD. This may have been a result of reduced power from premature study termination or from selection of steroid doses too low to suppress pulmonary inflammation adequately.

Several key points can be surmised from these trials: 1) Steroid therapy in the first week of life clearly has an unfavorable risk/benefit profile and therefore should be prohibited. 2) The concurrent use of indomethacin and steroids should also be avoided, 3) Outside of intestinal perforation, hydrocortisone therapy in the doses prescribed did not result in adverse effects typically associated with dexamethasone therapy, and 4)) Hydrocortisone in doses ≤ 2 mg/kg/day may not provide adequate anti-inflammatory activity to demonstrate modest reductions in BPD rates. Multiple proinflammatory cytokines such as interleukin (IL)-8 and transforming growth factor-β and chemotactic factors such as macrophage inflammatory protein-1 are present in the air spaces of ventilated preterm infants. These factors are found in higher concentrations whereas anti-inflammatory cytokines such as IL-10 are significantly reduced in infants who subsequently develop BPD.[54] This pro-inflammatory environment likely interferes with alveolar development as seen more commonly in very preterm infants in the post-surfactant era with the “new” form of BPD.[55] Higher doses have been associated with reduced need for extra oxygen in a small uncontrolled cohort of preterm infants treated with 5 mg/kg/day of hydrocortisone tapered over 22 days.[26] No short-term adverse effects typically seen with dexamethasone therapy were encountered with hydrocortisone treatment and no differences were found in neurodevelopmental outcome at 5 to 7 years as compared to a matched untreated cohort. In a different cohort of 60 preterm infants, 25 of whom were treated with a tapering course of hydrocortisone (5mg/kg/day for 33 median days), no differences were noted in the two groups at 8 years of age in relation to brain volumes on volumetric MRI or functional deficits as measured by standardized neurocognitive tests.[56] These reports all suggest that doses higher than 2 mg/kg/day are likely needed to control the inflammation associated with progressive BPD and that doses as high as 5 mg/kg/day may have beneficial pulmonary effects without any overt neurodevelopmental toxicity. These hypotheses are best tested in a well-conducted randomized trial with adequate measurement of early neurodevelopmental and pulmonary benefits as well as toxicity.

We hypothesized that hydrocortisone administration at a starting dose of 3 mg/kg/day tapered over 7 days (to ventilator-dependent high-risk ELBW infants) will improve total cerebral volume as measured by volumetric MRI by ameliorating pro-inflammatory cytokine mediated pulmonary and CNS injury. This dose is 2-3 times the basal production rate of cortisol and has been chosen as a “stress” dose with anti-inflammatory effects. This dose amounts to a cumulative exposure of 17 mg/kg of hydrocortisone. This is 3.5 mg/kg higher than the dose used in the Watterberg Trial.[23] An additional neurologic benefit of early treatment with hydrocortisone treatment at two weeks of age is that treated infants might be less likely to progress to severe BPD and receive higher dose

dexamethasone treatment. Such treatment may be neurotoxic not only because of the high dose but also presence of sulfite preservatives. For these reasons, we hypothesized that amelioration of BPD with hydrocortisone could ultimately improve rather than compromise long-term neurosensory outcomes in these infants. Before such a hypothesis is tested in a large-scale fashion, it is important to establish in a small cohort of ELBW infants the short-term therapeutic and adverse effects of such an approach. The measurement of total cerebral tissue by quantitative volumetric MRI may give us an early indication of therapeutic benefit or potential adverse effects of this previously unstudied hydrocortisone regimen.

3 STUDY DESIGN

This will be a single-center, phase II, randomized placebo-controlled, double-blinded trial with an intention-to-treat analysis comparing the 7 days of hydrocortisone (tapering dose of 3 mg/kg/day divided q 12 hours IV) to saline placebo administered between 10 and 24 days of life to a small group of ventilated ELBW infants (22 in each group) at high-risk for developing BPD. Consented eligible infants will be assigned randomly to receive either intravenous hydrocortisone or identical appearing saline placebo by a research pharmacist not involved in the patient's care or study recruitment. The clinical team, bedside nurses, and respiratory therapists will all be blinded to the treatment assignment. The only other changes to care of enrolled infants related to restrictions on the use of indomethacin for closure of patent ductus arteriosus and dexamethasone for BPD therapy. Due to concern for intestinal perforation, administration of indomethacin will be prohibited during the intervention period. Use of dexamethasone prior to 38 weeks PMA will be regulated and restricted to those infants older than 28 days of age on very high ventilator support (defined in more detail in section 5.3) and in doses of no greater than 0.2 mg/kg/day tapering doses for 7 days maximum. There will be no additional deviation from routine care and no laboratory evaluations required for study purposes only.

The primary outcome will be total cerebral tissue volume as measured by volumetric MRI at 38 weeks PMA. Our principal secondary outcome will be neurosensory impairments, including cognitive delay, cerebral palsy, deafness, and blindness at 18 to 22 months adjusted age. Additional outcomes to be evaluated include, death or disability, regional brain volumes, duration of mechanical ventilation, duration of oxygen (O_2) requirement, diagnosis of BPD (as assessed by O_2 therapy at 36 weeks PMA plus total O_2 duration ≥ 28 days with classification of severity) and survival free of BPD. Assessment of primary and secondary outcomes will be done in a blinded fashion. Neuroradiologists reading the MRIs, which are done already for clinical purposes in all ELBW infants, will be unaware of group assignment. Two of the investigators (NAP and REL) will do the quantitative MRI analysis also without knowledge of group assignment. Neurodevelopmental assessment at 18 to 22 months will be performed according to the NICHD Neonatal Research Network protocol with full masking of clinical history, cranial ultrasound findings, MRI findings, and study group assignment.

4 SELECTION AND ENROLLMENT OF SUBJECTS

In order to be randomized, an infant must meet all of the inclusion criteria and none of the exclusion criteria outlined below. Informed parental consent must be obtained prior to randomization.

4.1 Inclusion Criteria

4.1.1 Patient in the Memorial Hermann Children's Hospital (MHCH) NICU with a birth weight ≤1000g.

4.1.2 Ventilator-dependent between *10** and 21 days of age.

4.1.3 Respiratory index score (RIS: mean airway pressure x fraction of inspired oxygen) of ≥ 2.0 that is increasing or stable for the previous 24 hours or a RIS ≥ 3.0 if improvement noted in the past 24 hours.

Selection of a group of neonates at high risk for development of BPD appears critical in maximizing the benefit to risk ratio.[16] We elected to use a RIS cutoff based on work done by Papile and colleagues in a large cohort of very preterm infants[57] and validated in a NICHD Neonatal Network randomized trial of dexamethasone-treated preterm infants.[44] They demonstrated that a RIS > 2.4 in ventilated preterm infants on day of life 14 predicts BPD in 66% and death or BPD in 78% of such infants. We further validated this cut-off value in a cohort of ELBW infants cared for at MHCH. Over a 4 year period, from 1/1/1998 to 12/31/2001 there were 400 ELBW infants cared for at MHCH; 225 were still alive and on the ventilator at 14 days of life. Of the 225 ventilated babies, 206 (92%) had a RIS of ≥ 2.4. Of these 206 ELBW infants, 69.4% developed BPD (O2 requirement at 36 weeks PMA) and 86.9% had BPD or died before hospital discharge. In order to maximize enrollment, we chose a lower cut-off value of RIS (≥ 2.0) as this marker of illness predicted development of BPD (69.3%) or death/BPD (86.3%) comparably to an RIS of ≥2.4.

*The initial enrollment time period was 14 days, however 10 days of life is likely a better initial time point for recruitment for the following reasons: as the inflammation often begins in-utero in infants destined to develop bronchopulmonary dysplasia (BPD), initiating anti-inflammatory therapy such as hydrocortisone at an earlier time point may prevent additional damage to the developing lung. Meta-analysis of all steroid randomized trials has demonstrated that dexamethasone therapy initiated between 7 to 14 days of life leads to a greater BPD risk reduction (RR=0.62) as compared to therapy after 21 days of life.[40,41] Infants that later develop BPD often present with severe lung disease within the first week to two weeks of life as manifested by significant ventilator and oxygen dependence. Moving the target enrollment window to 10 days will allow us to target this population of infants and ideally prevent damaging fibrotic changes in the lung. The final enrollment time period will remain at 21 days of life.

4.2 Exclusion Criteria

4.2.1 Prior postnatal steroid treatment.

4.2.2 Evidence of sepsis or necrotizing enterocolitis.

4.2.3 Known major congenital anomalies of the cardiopulmonary or central nervous system.

4.2.4 Infants being treated with indomethacin or those likely to require treatment in the next 7 days as judged by the treating physician.

4.2.5 Inability or unwillingness of parent or legal guardian/representative to give written informed consent.

4.2.6 Gestational age <23 weeks.

4.3 Study Enrollment Procedures

4.3.1 Initial Screening: All new admissions to the MHCH NICU are already screened daily by a research nurse for the Volumetric MRI study (Specific Aim 1) to identify any ELBW infants. From this weekly updated list of candidates, the study coordinator and principal investigator will screen all infants that do not meet exclusion criteria. Between 10 and 21 days of life, these infants will be monitored daily for eligibility based on ventilator dependence and qualifying RIS. All attending neonatologists and unit respiratory therapists will be notified as well to identify eligible babies. An Eligibility Form will be completed for all ELBW infants that are admitted to the MHCH NICU during the enrollment period. Reasons for ineligibility will be documented clearly. If the infant is eligible, but not enrolled, reasons for nonparticipation will also need to be outlined in the form and the principal investigator notified immediately.

4.3.2 The study will not begin until IRB and NINDS approval are obtained. Written informed consent will be obtained from the infants' parents or legal guardians before the infant is eligible for enrollment in the study. The principal investigator or study coordinator (if principal investigator is treating physician) will obtain all informed consents. A sample informed consent form is included in Appendix I of this manual.

4.3.3 Once consent is obtained, the principal investigator or study coordinator will call the research pharmacist for group assignment. A research pharmacist not involved in the patient's care or study recruitment will randomly assign using sequentially numbered opaque sealed envelopes to receive either intravenous hydrocortisone or intravenous saline placebo based on the patient's weight measured that day. Randomization will be blocked and stratified by BW (≤ or >750 g) and by severity of lung disease (RIS 2.0 to 4 or > 4).

5 STUDY INTERVENTIONS

5.1 Interventions, Administration, and Duration

5.1.1 Randomized intervention

Hydrocortisone group: Hydrocortisone sodium succinate 1.5 mg/kg/dose q 12 hours (dexamethasone equivalent = 0.075 mg/kg/day) for days 1-4 followed by 1 mg/kg/dose days 5-6 and 0.5 mg/kg/dose on day 7, administered intravenously (IV).

Placebo group: Saline placebo (in equivalent volumes and identical color to hydrocortisone) q 12 hours for 7 days administered IV.

All study medication will be preservatives-free. For those infants on full feedings without an IV, one will be attempted. If an IV is not secured after three attempts, hydrocortisone or equivalent volume of saline placebo will be administered orally. All enrolled subjects will be treated in the neonatal ICU of MHCH. Although we do not anticipate any significant adverse events, based on previous studies with hydrocortisone, should adverse events occur, such as infections or hypertension, treatment will be at the discretion of the attending Neonatologist.

5.2 Concomitant Interventions

The treating physician has the option of withholding the study drug for up to 72 hours if an infant undergoes a sepsis evaluation or after consultation with the principal investigator, discontinuing it if the infant's clinical condition worsened. After the study period, corticosteroid use will be standardized. Our current nursery practice is to administer a 7-day weaning course of dexamethasone (0.5 mg/kg/day maximum starting dose) for infants with severe BPD causing respiratory failure. Since there is evidence and consensus that dexamethasone can improve respiratory function in infants with BPD.[45,58] withholding "rescue" steroid therapy from infants with severe respiratory failure likely to die from BPD is deemed to be unethical by many neonatologists. We will therefore allow its use in both our treatment arms when infants progress to severe BPD. However, to standardize its use and maintain consistency with recommended guidelines and current nursery practice, we will limit consideration of dexamethasone treatment to study infants ≥ 28 days of age. Additionally, the following respiratory failure criteria will need to be met: RIS > 10 for at least 24 hours (predicted 1 year mortality 38.5% for all 400 ELBW infants cared for at Hermann Hospital between January 1998 and December 2001-data derived from NICU Database). Clinicians will be encouraged to use lower doses of dexamethasone so as to minimize adverse effects of this potent medication.

The maximum dose and duration of dexamethasone (or equivalent dose of another corticosteroid) permitted for trial participants will be as follows: 0.2 mg/kg/day x 3 days, followed by 0.1 mg/kg/day for the next 4 days. All doses are divided q 12h and given IV or PO. This dose has been shown to be comparable to the more commonly used 0.5 mg/kg/day dose in improving respiratory parameters.[37] If relatively early treatment with hydrocortisone is effective in reducing the severity of BPD, fewer infants in the hydrocortisone group will receive "rescue" corticosteroids as compared to infants in the control group (and as compared to similar non-study infants).

During study drug administration clinicians will be encouraged to closely follow respiratory status, including chest wall rise, tidal volumes, oxygen saturations, chest x-rays and blood gases and provide appropriate pressures and oxygen as is done commonly for all ventilated infants. All study infants will otherwise be managed according to nursery routines for ELBW infants. The current nursery practice is to maintain oxygen saturation in the 85-95% range. This practice was recently modified based on studies suggesting that higher O_2 saturation ranges might cause worse lung disease[59] and do not result in better neurologic outcomes.[60] Serum $PaCO_2$ values >50 are allowed if a pH >7.20-7.25 is maintained.[61] Blood glucose monitoring is generally done every morning with daily electrolyte checks as part of routine care for ill infants. Blood pressure monitoring is done every 4-6 hours in the NICU as per routine for all sick patients. Blood glucose monitoring is generally done every morning with daily electrolyte checks as part of routine care

for ill infants; if serum or bedside glucose monitoring is not being performed for clinical purposes, we will recommend a bedside glucose test once a day while the patient is receiving the study intervention. Screening for intracranial hemorrhage and white matter injury with cranial US is performed at a minimum once at 10-14 days for all ELBW infants.

5.3 Adherence Assessment

Compliance should not be an issue in our population of newborn infants; however our research nurses will record the number of formulations of the study drug that were administered. Protocol violations will be recorded and the principal investigator will be notified immediately. After the study code is broken, correct assignment of hydrocortisone and placebo per randomization assignment will also be assessed.

6 CLINICAL AND LABORATORY EVALUATIONS

The study does not require any laboratory or clinical investigations which are not already part of routine care. The study coordinator will follow enrolled patients daily while they are receiving the study drug and for an additional 3 days thereafter. During this 10 day period, daily blood gases, glucose levels, systolic and mean blood pressure, and ventilator settings (PIP, PEEP, Ti, IMV, FiO_2) will be recorded. Medication logs will be checked to ensure twice daily administration of study drug and route of administration. Any adverse events noted in the nursing or physician records will be recorded and the principal investigator notified.

All brain MRIs in ELBW infants performed at 38 weeks PMA are already being transferred (as part of the Volumetric MRI Study-Aim 1 of K23 grant) to a workstation in the principal investigator's lab for post-image processing to generate 3-D volumes.

7 MANAGEMENT OF ADVERSE EXPERIENCES

Based on the three randomized studies and one uncontrolled study discussed earlier,[22,23,26,51] it is evident that hydrocortisone administration results in far fewer adverse events as compared to dexamethasone administration in this population of ELBW infants. Nonetheless, strict precautions are in place to detect all adverse events and respond appropriately if and when side-effects occur:

1. Gastrointestinal bleeding/gastritis – This adverse event has not been noted in hydrocortisone treated infants. The research nurse will document daily any occurrence of GI bleeding/gastritis. Management will be per discretion of the clinical team. GI bleeding/gastritis in dexamethasone trials generally has been limited to a small amount of bleeding that has not been profound enough to result in study drug discontinuation.

2. Hyperglycemia – Hyperglycemia or insulin therapy for hyperglycemia has not been noted with increased frequency as compared to placebo treated patients in the four trials mentioned earlier, including the trial which used 5 mg/kg/day of hydrocortisone. Daily blood glucose monitoring that occurs for clinical purposes in most infants in our nursery at 2-3 weeks of life will provide monitoring for this potential adverse event. If hyperglycemia does occur, therapy with insulin will be up to the discretion of the clinical team. The research nurse will document the daily blood glucoses for all enrolled infants.

3. Hypertension - Cuff blood pressure will be monitored every 4 hours while the patient is receiving the study drug and recorded daily by a research nurse. If the systolic blood pressure (SBP) if between 2 and 3 standard deviations (SD) for age for >24 hours, the dose of the medication will be reduced in half and the BP will be monitored more frequently. If the blood pressure remains elevated, the study drug will be discontinued. For infants will SBP > 3 SD for >24 hours, the study drug will be discontinued. Increased risk of hypertension has not been noted in any of the hydrocortisone studies mentioned earlier.

Treatment for persistent elevation of BP will be at the discretion of the attending Neonatologist however the following proposed treatment regimen will be suggested*:

- If SBP is between 2 and 3 SD for age for > 24 hours, treat with an initial dose of Hydralazine 0.1 mg/kg/dose IV q 8 hours and titrate up as needed until the SBP is < 2 SD for age.
- If SBP > 3 SD for age for > 24 hours, initiate Hydralazine at 0.3 mg/kg/dose IV q 8h and titrated up as needed until SBP is < 2 SD for age.

*The maximum dose for Hydralazine should not exceed 2 mg/kg/dose. Once the SBP is < 2 SD Hydralazine administration should be made prn for SBPs > 2 SD only.

4. Infections - Patients are already monitored closely by the clinical team for increased risk of infections as this is a high-risk population for infections as it is. A research nurse will monitor and document the progress of all infants daily while receiving the intervention. If an infant is suspected to have a clinical infection/sepsis, it is up to the discretion of the attending Neonatogist if they want to discontinue the study drug. Increased risk of infections has not been noted in any of the hydrocortisone studies mentioned earlier.

5. Intestinal perforation or necrotizing enterocolitis (NEC) - If a patient is suspected of having an intestinal perforation, a stat KUB and left lateral decubitus X-ray of the abdomen will be performed to rule out free air. In the presence of free air, the study drug will be discontinued immediately, and the surgeon notified stat. Within 24 hours, the principal investigator will also notify the UT-Houston IRB and GCRC, as well as the study IMM and NINDS. If medical NEC is suspected, the study drug may be discontinued by the attending Neonatologist if they so choose. Increased risk of NEC has not been reported with hydrocortisone administration. As discussed earlier, although intestinal perforations have been noted with increased frequency in hydrocortisone treated preterm infants, this risk was primarily seen in infants also concurrently treated with indomethacin primarily within the first week of life. By initiating hydrocortisone after 2 weeks of life and restricting the use indomethacin, we do not anticipate an increased risk on intestinal perforations for subjects enrolled in this trial.[52]

8 CRITERIA FOR INTERVENTION DISCONTINUATION

The study drug will be discontinued for the following reasons only:

1. Persistent hypertension as defined earlier in section 7.
2. Intestinal perforation.
3. A hemodynamically significant PDA causing left heart failure that requires immediate indomethacin therapy per attending Neonatologist.
4. Attending Neonatologist request if patient develops an infection or NEC.
5. Parental wish to withdrawal their infant from the study.

All subjects that have been withdrawn for any reason will still be followed to 18 to 22 months adjusted age for neurodevelopmental and general health assessment. Irrespective of weather treatment was completed, these infants will still be analyzed in their own randomized group.

9 STATISTICAL CONSIDERATIONS

9.1 General Design Issues

This will be a single-center, phase II, randomized placebo-controlled, double-blinded trial with an intention-to-treat analysis comparing the 7 days of hydrocortisone to saline placebo administered between 10 and 21 days of life to a small group of ventilated ELBW infants (22 in each group) at high-risk for developing BPD. We hypothesized that among ELBW infants at high risk for BPD and neurologic impairments, those infants randomized to seven days of hydrocortisone will demonstrate increased total cerebral tissue volumes as compared to infants randomized to placebo. We further hypothesize that hydrocortisone treatment will result in a decreased risk of neurosensory impairments as measured by standardized blinded examiners at 18 to 22 months corrected age. Measurement of total and regional cerebral volumes will be done by manual and semi-automated segmentation of 3-D MRI images performed at 38 weeks PMA while masked to treatment assignment, study data, and results of previous neuroimaging findings. We have already developed a standardized validated method for measuring 3-D brain volumes.[36,62] Measurement of neurosensory impairments, including cerebral palsy, cognitive impairments, and hearing and vision deficits is already done in a standardized and validated fashion in our high-risk clinic while fully masked to clinical history, neuroimaging findings, and study group assignment; we are one of the 16 NICHD Neonatal Research Network centers that follow all ELBW infants and tests their neurodevelopmental status at 18 to 22 months corrected age. The length of follow-up was in part chosen by this already offered service. Ideally if we obtain further funding we will seek to follow these infants to at least 5 years of age to improve our ability to detect meaningful differences in neurocognitive outcomes.

Stratification by birth weight (BW ≤750g and BW>750 -1000g) and degree of lung disease (RIS score 2 to 4 and RIS score >4) because these factors are clearly confounders that can significantly alter the relationship of treatment assignment to our chosen outcomes. Therefore to minimize group imbalance, we have chosen to stratify our two study groups into these 4 strata.

9.2 Outcomes

9.2.1 Primary outcome

The total cerebral tissue volume (combined gray and white matter, no CSF) as measured by volumetric MRI at 38 weeks PMA.

9.2.2 Secondary outcomes

a. Neurologic

1) Neurosensory impairments at 18-22 months adjusted age (defined below); 2) Cerebellar volume; 3) Subcortical gray matter; 4) Total gray matter; 5) Total white matter; 6) Total cortical surface area and complexity;[63] and 7) Abnormalities on anatomic MRI.

b. Pulmonary
1) Survival free of BPD at 36 weeks as assessed by: O_2 therapy at 36 weeks PMA plus total O_2 duration ≥ 28 days with classification of severity[64,65] and defined as O_2 therapy at 36 weeks PMA; 2) Respiratory index score on days 3 and 7 of intervention and 1 week post-intervention; 3) Subsequent need for dexamethasone therapy for worsening BPD; 4) Total duration of mechanical ventilation; 5) Total duration of O_2 supplementation; 6) Total duration of hospital stay; 7) Discharge home on O_2 therapy and 8) Total duration of hospital stay prior to 18 months of age.

c. Other
1) Death or neurosensory impairment at 18-22 months adjusted age; 2) Growth as assessed by weight and head circumference at 38 weeks PMA; 3) Bacteremia > 48 hours after randomization as defined by a positive blood culture treated with antibiotics for at least 7 days; and 4) Sepsis as defined by a positive blood culture, CSF culture, and/or strong suspicion of infection per clinical team resulting in antibiotic treatment for minimum of 7 days.

9.3 Sample Size and Accrual

We will test the primary hypothesis by comparing the total cerebral tissue volumes (combination of total gray and white matter) in the hydrocortisone treated and placebo treated study groups. In the non-randomized cohort study by Murphy et al,[34] the total cerebral tissue volume was 312 ± 44 mL in the dexamethasone group and 448 ± 50 mL in the non-dexamethasone group, corresponding to a 6-week delay in cerebral growth. Because this was a non-randomized study with sicker and smaller babies in the dexamethasone group as compared to the unmatched comparison group, we are proposing that two-thirds (67%) of the effect size in this study was explained by the effects of BPD (a known independent risk factor for cognitive and motor delays) or other confounding group differences and one-third (33%) of the difference was the result of adverse neurotoxic effects of dexamethasone. Using this estimation, the total cerebral tissue volume at term PMA for a premature infant with BPD untreated with dexamethasone should approximate 357 mL, equivalent to a 4-week delay in cerebral growth. We are powering our study to detect a 2 week difference (43 mL) in total cerebral tissue sparing effect in hydrocortisone treated infants with evolving BPD over similar placebo treated infants. This will allow for a conservative estimate of sample size that will still be powered to test our primary hypothesis despite likely dexamethasone use in groups.

Assuming a type I error of 0.05 and a known estimated standard deviation of 50 mL, we would need 22 premature infants with evolving BPD in each group to achieve 80% power to detect a 43 mL difference in total cerebral tissue volumes between the two treatment groups using a two-sided test. (Note: The estimated sample size does not change despite one interim look halfway into enrollment - tested using the O'Brien-Fleming spending function in NCSS). In this very preterm cohort of ELBW infants, we estimate an approximately 20% mortality rate prior to primary outcome determination at 38 weeks PMA. We will therefore need to enroll 28-30 infants per group (22/0.8) to determine the primary outcome in at least 22 infants. Over a 4 year period, from 1/1/1998 to

12/31/2001 there were 400 ELBW infants cared for at MHCH; 212 (53%) were still alive and on the ventilator at 14 days of life with a RIS of ≥ 2.0.

In 2004, 129 ELBW infants were admitted to Memorial Hermann Children's Hospital's NICU (only one infant < 23 weeks gestational age). Based on our own institution's data, from 1998-2001, 53% of all ELBW infants are ventilator treated at 14 days after birth with a respiratory index score of ≥ 2.0 (see Sample size estimation section of protocol for more details). We estimate another 7% will become ventilator dependent between 15 and 21 days of life. That would give us an eligible pool of 77 ELBW infants (129 x 0.6). The percentage that would meet criteria for exclusion from our study is likely to be no greater than 30%. That would result in an **annual enrollment rate of 54 eligible infants** (77 x 0.7). Even with a more conservative estimate of 40%, we would still enroll 46 study patients annually (77 x 0.6). Given a 20% mortality rate prior to primary outcome determination at 38 weeks PMA, we will therefore need to enroll 28-30 infants per group (22/0.8) to determine the primary outcome in at least 22 infants. Therefore, based on our estimate of percent exclusion rate, **enrollment should be complete within 13 to 16 months**. Even if our estimates are conservative and enrollment takes longer, it will still be within the timeframe of the K23 grant. We recognize that this sample size will not provide adequate power to meaningfully assess the secondary outcomes. But information on these secondary outcomes will be useful for planning future studies and could be combined with data from future studies in meta-analyses.

9.4 Data Monitoring

Based on our high-risk population, the low risk nature of our intervention, and NINDS Guidelines on Data and Safety Monitoring, we have chosen an Independent Medical Monitor (IMM; pending NINDS approval), Dr. Waldemar Carlo, of the University of Alabama (contact information below). Dr. Carlo is an experienced researcher and international authority in the field of neonatal lung diseases.

Dr. Carlo will receive a study update from the principal investigator on a quarterly basis, including the number of subjects screened and enrolled, drop-outs, all adverse events, and primary and secondary efficacy endpoints achieved. For unexpected serious adverse events (i.e., intestinal perforations) you will be notified within 24 hours. At each monitoring interval, Dr. Carlo will update Dr. Hirtz, the NINDS Program Director that he has reviewed the ongoing study activities with emphasis on data integrity, protocol adherence and enrolled participant safety issues. This will especially include the review of adverse events and reasons for losses to follow-up, raising any concerns or issues with the NINDS and the principal investigator.

There will be a blinded interim analysis of all the data halfway into enrollment (total of 28 patients). If in Dr. Carlo's opinion there are significant concerns regarding drug safety, he will select two additional faculty members with expertise in neonatal lung disease and analysis of clinical trials to assist him in making a recommendation as to the continuation, modification, or conclusion of the trial. There will be no separate monitoring considerations for each stratum of the study population due to the small sample anticipated in each stratum (<8 infants per stratum for each study group). Due to the small size of our trial it is highly unlikely that a significant benefit or harm will be detected with only 28 infants and therefore no stopping rules are being defined apriori.

Waldemar Carlo, MD
Division Director, Neonatology
University of Alabama
525 New Hillman Building
UAB Station
Birmingham, AL 35294
205-934-4680 Voice
205-034-3100 Fax
wcarlo@peds.uab.edu

9.5 Data Analyses

An intention-to-treat analysis will be performed. The primary analysis of total absolute cerebral tissue volume will be an analysis of covariance (ANCOVA) adjusting for PMA at assessment. An analysis of standardized brain volume (e.g., the total cerebral tissue volume divided by the total intracranial volume) will also be performed to adjust for differences in cranial size. The distributions of potentially important confounding variables at baseline (BW, GA, gender, race, antenatal steroids, Apgar scores) will be compared in the two groups using 2-sample t-tests or Wilcoxon-Mann-Whitney tests for continuous outcomes and Chi-squared or Fisher's exact tests for categorical outcomes. If differences exist, secondary analyses using multiple regression models will be performed to adjust for these differences. Since BW and degree of respiratory illness are potential confounders, the planned stratification at study entry should balance out the two groups in relation to these two covariates. Dexamethasone therapy will not be considered as a confounder because a reduction in its use could be part of the causal chain by which hydrocortisone results in better brain volumes. Consistent results from the adjusted and unadjusted analyses will support the study conclusions.

Secondary outcomes will be assessed using T-tests or rank order tests to test the secondary hypotheses for continuous dependent variables, Chi-square tests or Fisher exact tests for categorical variables or Kaplan Meier analysis for time to event outcomes (e.g., survival free of BPD). If confounding variables are identified based on group differences at baseline, secondary analyses will be performed using linear regression for continuous outcomes, logistic regression for categorical outcomes, or Cox proportional hazards regression for time to event outcomes. A beneficial or detrimental effect of hydrocortisone based on gender or racial/ethnic subgroups has note been observed in previous trials and therefore a subgroup analysis is not planned.

The occurrence of premature deaths prior to primary outcome assessment at 38 weeks and secondary outcome assessment at 18 to 22 months is an unfortunate reality in this high-risk population of infants. Several approaches can be adopted to statistically deal with this problem. One could exclude these infants from all analyses and perform analyses only on survivors that have measured outcomes to analyze. This could be problematic however, if intervention influences mortality and there are significant differences in survival between the groups. In such a situation, assigning the worst outcome to those that die may be an acceptable solution. For example, all infants that die after discharge home but before 18 to 22 months neurosensory assessment can be assumed to have neurosensory impairment and assigned this poor outcome. This approach would allow us to potentially preserve informative data.

Poor follow-up rates can also lead to missing data and result in unreliable results. Extensive effort and expense is devoted to ensuring excellent follow-up rates in our high-risk clinic. Our clinic physicians and follow-up coordinators, in addition to establishing strong relationships with the families of ELBW infants, take extra measures such as frequent telephone contacts and in-home visits when necessary to ensure adequate follow-up of these high-risk infants. Despite having a large population of ELBW infants, our center has annually consistently followed up greater than 80-90% of survivors at 18 to 22 months adjusted age. Due to recent efforts initiated by the principal investigator to further increase the frequency of contacts and follow-up services to our ELBW infants, we are expecting a 100% follow-up rate for infants enrolled in the Hydrocortisone Trial.

10 DATA COLLECTION, MONITORING, AND ADVERSE EXPERIENCE REPORTING

10.1 Records to Be Kept

Data collection will be minimized to relevant information necessary in determining safety and efficacy as defined by our primary and secondary end-points and protocol adherence. If additional information is required later, it will be extracted for the NICHD network GDB data forms collected on all ELBW infants at center. Patient confidentiality will be maintained throughout the study. Access of the clinical information and MRI images will be limited to the four investigators, a research nurse and a research technician. Once clinical information and MRI images of the brain are obtained and linked for the study individuals, a unique study identifier will be assigned to each patient to replace all unique identifiers. Prior to de-identification, data will be stored in password-protected files on a PC hard drive in a locked office. No portable devices will be used. When storage space on a secure university server is made available, data will be stored there instead of on the local hard drive. Protected information will be stored securely and then removed from the data files as soon as feasible.

10.2 Role of Data Management

The principal investigator is responsible for the following data collection and management activities:1) Developing, printing and distributing the data collection forms, including periodic updates as necessary; 2) Setting up a Microsoft Access™ database, ensuring that data are correct and complete by implementing editing and auditing procedures; 3) Monitoring the progress and quality of the study; 4) Reporting adverse events promptly to the IMM, IRB, NINDS, and GCRC; 5) Provide study updates to the IMM on a quarterly basis and the IRB, GCRC, and NINDS on an annual basis; 6) Perform all data analysis; and 7) Assuring patient confidentiality.

The research coordinator is responsible for the following data collection and management activities: 1) Collecting and entering all required data in a timely and accurate manner; 2) Maintain confidentiality with patient health data; and 3) Notifying the principal investigator promptly when adverse events occur.

10.3 Adverse Experience Reporting

Adverse events are to be recorded on the Adverse Event Form (see Appendix II) and reported to the principal investigator, Dr. Parikh immediately. Serious adverse events will be communicated to the local IRB and study IMM within 24 hours. Reporting of all other adverse events will take place within 5 days to the IRB and on a quarterly basis to the

study IMM, unless many adverse events are noted or the IRB stipulates a more frequent interval of adverse event data analysis. Additional details on the definition of the adverse events will be provided separately.

11 HUMAN SUBJECTS

11.1 Institutional Review Board (IRB) Review and Informed Consent

This protocol and the informed consent document (Appendix I) and any subsequent modifications have been reviewed and approved by the UT-Houston IRB. A signed consent form will be obtained from a parent or legal guardian. The consent form describes the purpose of the study, the procedures to be followed, and the risks and benefits of participation. A copy of the consent form will be given to the subject, parent, or legal guardian, and this fact will be documented in the subject's record.

11.2 Subject Confidentiality

All laboratory specimens, evaluation forms, reports, video recordings, and other records that leave the site will be identified only by the Study Identification Number (SID) to maintain subject confidentiality. All records will be kept in a locked file cabinet. All computer entry and networking programs will be done using SIDs only. Clinical information will not be released without written permission of the subject, except as necessary for monitoring by IRB, the GCRC, the study IMM, and the NINDS.

11.3 Study Modification/Discontinuation

The study may be modified or discontinued at any time by the IRB, the NINDS, the study IMM, the OHRP, the FDA, or other government agencies as part of their duties to ensure that research subjects are protected.

12 PUBLICATION OF RESEARCH FINDINGS

Publication of the results of this trial will be governed by the policies and procedures developed by the Study Committee. Any presentation, abstract, or manuscript will be made available for review by the sponsor and the NINDS prior to submission.

APPENDIX I

SAMPLE INFORMED CONSENT FORM

TITLE: A Randomized Trial of Hydrocortisone in Very Preterm Infants at High Risk for Neurologic and Pulmonary Impairments (CPHS HSC-MS-05-0218)

Principal Investigator: Dr. Nehal A. Parikh, University of Texas - Houston Medical School

You are being invited to allow your baby to take part in a research project called the "Hydrocortisone Trial" being conducted by Dr. Nehal Parikh at Memorial Hermann Children's Hospital. You should understand enough about the risks and benefits of this study to make an informed decision on enrolling your baby. Your decision to take part is voluntary and you may refuse to take part, or choose to stop taking part, at any time. A decision not to take part, or to stop being a part of the research project will not change the services that your baby receives from the hospital and its doctors.

This consent form gives information about the research study which will be discussed with you in greater detail. Once you understand the study, you will be asked to sign this form if you want your baby to take part in this study.

This research project has been reviewed by the Committee for the Protection of Human Subjects (CPHS) of the University of Texas Health Science Center at Houston as HSC-MS-05-0218.

RESEARCH PURPOSE: The purpose of this study is to assess the effects of lung disease prevention by using a drug called hydrocortisone by measuring brain volumes of enrolled infants prior to discharge home. Your premature baby needs a breathing machine and extra oxygen due to underdeveloped lungs resulting from early birth. Compared to other premature babies not requiring a breathing machine, your baby is at a higher risk of remaining on the breathing machine and extra oxygen for a long time. Babies that require a breathing machine and extra oxygen for several weeks develop injured lungs that put them at higher risk for increased infections, poor growth, abnormal muscle tone, and abnormal brain development, as they get older. Although your baby is at higher risk, it is not certain that your baby will have these problems. Dexamethasone, a steroid medication reduces the risk of such problems by stopping some of the harmful lung injury. We no longer use this medication often due to the many side-effects we

now know it causes, such as abnormal muscle and brain development. Only those babies that are older and very sick (more severe than your baby's condition) are treated with dexamethasone. At present, there are no therapies for this problem early in life.

The doctors at Memorial Hermann Children's Hospital are taking part in a study to see if earlier use of a less powerful steroid medicine called hydrocortisone will also help stop lung injury but cause less harm that the stronger dexamethasone. A medicine that has no side-effects on the brain and improves lung disease should allow babies and their underdeveloped brains to grow better and offer greater chances of developing normally as they get older. This study therefore will compare the effects of hydrocortisone to infants untreated with any similar medications.

RESEARCH PROCEDURES: If you agree to have your baby take part in this study, he/she will be randomly chosen (like the flip of a coin) to receive hydrocortisone or a saline placebo (salt water). This will be given into the stomach through a feeding tube already in place for babies on full feedings, or into your baby's veins through an already existing catheter. In order to obtain the most accurate results, the research pharmacist will be the only person with knowledge of which babies received hydrocortisone and which ones received the saline placebo. The medication or saline placebo will be given two times a day, for a total of 7 days. Throughout the study period and until hospital discharge, your baby will be monitored closely, similar to other babies in the intensive care nursery.

As part of routine care, a magnetic resonance imaging (MRI) scan of the brain, a specialized study that takes detailed pictures of the brain, is done on all very preterm babies for clinical purposes whether or not they are part this study. This routine MRI will be obtained by the study investigators to further evaluate the effects of hydrocortisone on the size of the brain. After your baby is discharged from the hospital, he/she will be seen in the follow-up clinic at 18 months after discharge, as part of routine care offered to all very premature babies to evaluate their motor and brain function. The clinic visit will include an interview, a physical examination, and a test that will check your baby's brain and motor development.

TIME COMMITMENT: Agreement to take part in this study should not increase the time you need to devote to your baby's care. The main difference in care your baby will receive will be receiving the study drug by a nurse for seven days. This will not increase the total hospital stay for your baby. We routinely follow all premature babies like yours for up to 18 months after dis-

charge in our High-Risk Follow-up Clinic for clinical purposes. You will need to maintain appointments for follow-up of your baby until at least 18 months after discharge so we can see how your baby's brain and lung function developed after taking part in this study.

POSSIBLE BENEFITS: Your baby may receive no direct benefit from being in this study; however, by allowing your infant to take part in the study it may help other infants get better care in the future. The possible benefits of hydrocortisone in high risk babies such as yours may be improved brain growth and reduced need for the breathing machine and extra oxygen. This may in turn improve long-term brain function development and reduce rates of infections after hospital discharge. Your baby could also benefit from closer monitoring that is part of the research project. Additionally, knowledge gained from this study may help in the treatment of babies born in the future.

POSSIBLE RISKS: Previous studies with hydrocortisone at lower doses have shown minimal side-effects. The main side-effect noted was a higher risk of developing a small hole in the intestines (gut). This risk however was seen mainly in babies who were less than 2 weeks old and in those that received hydrocortisone with a drug called indomethacin. Indomethacin is a drug that is used in premature babies to close an open blood vessel called a ductus arteriosus. Your baby is not on indomethacin now and your doctor will not start indomethacin during the next seven days when the hydrocortisone or saline placebo will be given. The effects of hydrocortisone on brain growth and long-term brain function are unknown and a small possibility of harm to the brain cannot be **excluded**. Treatment with dexamethasone (a stronger steroid medication) in the first week of life can result in an increased risk of **delayed development**. Treatment after the first week of life in higher risk infants, such as in this trial, has not been shown to increase rates of delayed brain development. There also may be risks involved that are not known to researchers at this time.

ALTERNATIVES: You may select other options than agreeing to enroll your baby in this research study. If you choose not to take part, your baby's doctor will ultimately decide if and when steroids are appropriate treatment for your baby.

STUDY WITHDRAWAL: You may take your baby out of this study at any time. If you decide to do this, your baby will continue to receive the same care he/she would if they were never in the study. The hospital and your doctor will continue to provide treatment with oxygen, breathing machine support and other medications as needed.

CONFIDENTIALITY: Information about what the doctors learn from this study may be published or given to other people doing research, but neither your name nor your baby's name will be used. The data collected in this study may be submitted to the National Institutes of Health which is sponsoring this study. You or your baby will not be personally identified in any reports or medical articles that may result from this study. Any personal information gathered on you or your baby as part of this study will be part of his/her medical record and will otherwise be kept confidential. A special number will be used to identify your baby in the study and only the investigator at Memorial Hermann Children's hospital will know his/her name.

FINANCIAL COSTS: You will not receive payment for taking part in this study nor is there a cost to you for taking part in this study. Your insurance company, another third party payer, or you will be responsible for payment for treatments that are considered "routine care". "Routine care" means treatments your baby would receive even if he/she were not taking part in this research study, such as medications, diagnostic studies, and follow-up visits.

IN CASE OF INJURY: If an injury occurs, you will report this injury to the principal investigator, Dr. Nehal Parikh (713-990-1040) and to the Committee for the Protection of Human Subjects at (713) 500-3985. If your baby suffers any injury as a result of taking part in this research study, you understand that nothing has been arranged to provide free treatment of the injury or any other type of payment. However, all needed facilities, emergency treatment and professional services will be available to your baby, just as they are to the community in general. You will report any such injury to Dr. Parikh at 713-990-1040 and to the Committee for the Protection of Human Subjects at 713-500-3985. You will not give up any of your legal rights by signing this consent form.

QUESTIONS: Any questions you have asked about this study have been answered to your satisfaction. If you have any further questions later on, Dr. Nehal Parikh at 713-704-2900 will be glad to answer your questions at any time.

By signing this paper, you are saying that you have read it, understand it, and that you agree for your baby to take part in this study.

Signature of Legally Authorized Representative	Date	Time
Relationship to Subject	Date	
Signature of Witness	Date	Time
Signature of Investigator/Designee Obtaining Informed Consent	Date	Time

This study **(HSC-MS-05-0218)** has been reviewed by the Committee for the Protection of Human Subjects (**CPHS) of the University of Texas Health Science Center at Houston**. For any questions about research subject's rights, or to report a research-related injury, call the CPHS at (713) 500-3985.

APPENDIX II

SAMPLE ADVERSE EVENT FORM

**Protocol Number**	**HSC -MS - 05 - 0218**
**Full Protocol Title:**	**A Randomized Trial of Hydrocortisone in Very Preterm Infants at High Risk for Neurologic and Pulmonary Impairments**
**Principal Investigator:**	Nehal A. Parikh, DO
**Date of Adverse Event:**	
**Was Adverse Event:**	☐ Expected ☐ Expected but at a higher frequency<br>☐ Unexpected
**Type of Report:**	☐ Initial ☐ Follow-up
**Does this AE involve your subject?**	☐ Yes (local) ☐ No (other site)
**Is this AE being reported within 5 working days of its occurrence?**	☐ Yes ☐ No*<br><br>*If no, attach a detailed explanation as to why the adverse event was not promptly reported to the CPHS.

**Did event involve or result in:**	**Description of Adverse Event:**
☐ **Death**<br>☐ **Gastrointestinal bleeding/gastritis**<br>☐ **Hypertension**<br>☐ **Hyperglycemia**<br>☐ **Intestinal perforation**<br>☐ **Necrotizing enterocolitis**<br>☐ **Sepsis**<br>☐ **Medical or surgical intervention**<br>☐ **Increase in level of care**<br>☐ **Prolonged hospitalization**<br>☐ **Protocol Deviation (including overdose)**<br>☐ **Persistent or significant incapacity/disability**	

**Severity of AE:**<br>☐ Grade 1	**MILD** - Transient or mild discomfort; no limitation in activity; no medical intervention/therapy required
☐ Grade 2	**MODERATE** - Mild to moderate limitation in activity, some assistance may be needed; no or minimal medical intervention/therapy required
☐ Grade 3	**SEVERE** - Marked limitation in activity, some assistance required; medical intervention/therapy required, hospitalization possible
☐ Grade 4	**LIFE THREATENING** - Extreme limitation in activity, significant assistance required; significant medical intervention/therapy required; hospitalization or hospice care probable

**Follow-Up**<br>If this adverse event involves your subject **at your site**, please indicate whether this event:<br><br>☐ Is not resolved and requires follow-up<br><br>☐ Is resolved and does not require follow-up	If this adverse event involves your subject **at your site**, please indicate whether the subject is:<br><br>☐ Permanently off of the study protocol<br><br>☐ Has already completed the study<br><br>☐ Remains on the study
Has this type of adverse event occurred previously?	☐ Yes* ☐ No *If yes, please provide incidence data:
Did the subject receive medical treatment or increased medical treatment as a result of this event?	☐ Yes* ☐ No *If yes, include explanation:
Does your protocol have a Data Safety Monitoring Board?	☐ Yes* ☐ No<br><br>*If yes, have you or will you notify the DSMB?<br>☐ Yes ☐ No*<br>If no, please explain:
Was the AE a result of a protocol deviation?	☐ Yes* ☐ No *If yes, include explanation:
**Assessment of Cause**:<br><br>In the **judgment of the sponsor**, what is the likelihood that the event was related to the study:	☐ Definitely ☐ Probably<br><br>☐ Possibly ☐ Not related ☐ Unknown<br><br>☐ No assessment provided by sponsor

In the **judgment of the principal investigator**, what is the likelihood that the event was related to the study:<br>☐ Definitely ☐ Probably<br>☐ Possibly<br>☐ Not related ☐ Unknown	**Provide brief rationale for decision:**
In the judgment of the **principal investigator**, does this development **indicate an increased risk** for currently enrolled subjects OR future subjects?	☐ Yes* ☐ No *If yes, include explanation:
In the judgment of the **principal investigator**, should the study protocol be changed as a result of this AE?	☐ Yes -> Include change request for amendment to protocol.<br>☐ No -> **Justify why amendment to protocol is not necessary:**
In the judgment of the **principal investigator**, should the currently approved informed consent form be revised as a result of this adverse event?	☐ Yes -> Attach a copy of the new form for review and approval.<br>☐ No -> **Justify why changes to ICF are not necessary**:
In the judgment of the **principal investigator**, should currently enrolled subjects be notified of this event and/or be re-consented?	☐ Yes -> Attach method and details of contact/re-consent.<br>☐ No **->** **Include justification as to why subjects do not have to be notified:**

Statement of Principal Investigator:
"I have personally reviewed this report and agree with the above assessment."

______________________________ __________________
Signature of Principal Investigator Date

______________________________ ______________________________
Printed name of preparer Signature of preparer

Reminder: *Enter this AE on the Adverse Event Log which is required to be submitted annually at the time of Continuing Review.*

APPENDIX III

Magnetic Resonance Imaging Protocol

As per the nursery routine, an anatomical MRI will be routinely obtained at 38 weeks PMA or, if discharged sooner, prior to discharge for all ELBW infants. If an MRI cannot be successfully performed at this time, a scan as close to this time as possible will be performed. Postmenstrual age will be determined as precisely as possible using the best obstetric estimate of GA at birth. The obstetric rather than pediatric estimate of GA is used because of evidence of both systemic and random error in pediatric estimates.[66]

The neonatal brain has relatively higher water content as compared to children and adults. MRI sequences at MHCH have been specifically tailored for the neonate with higher T2 relaxation times with resultant improvements in signal to noise ratio.[67],[68] ELBW infants are routinely imaged in a single scanner (GE LX) with a protocol that begins with a multi-planar localizer scan (estimated time ~1 minutes), followed by T1-weighted slices in all 3 planes (~6 min), coronal T2 slices (~ 2 min), T1-weighted 3D echo spoiled gradient (SPGR) multi-slice scan (~ 5 min), and finally, a double echo PD/T2 weighted Fast/Turbo spin echo scan (~ 3 min). The first scan is a quick scan to orient the subsequent scans and set parameters. The last three scans are critical for our proposal and will be used for the quantitative studies. If necessary, these three scans will be repeated until they are acquired satisfactorily to ensure reliable measurement. The PD/T2 weighted scan provides better resolution for quantitative studies in infants less than a year old. The table below has the acquisition details for the scans.

A single neuroradiologist will read all MRI scans for clinical evidence of CNS pathology. Definition of an abnormal brain scan has been standardized. Cerebral volumes will be generated utilizing advanced post acquisition techniques utilizing the Analyze software developed by Mayo Clinic (Biomedical Imaging Resource, Mayo Foundation, Rochester, Minn) and commercially available for research purposes.[69] Several different brain regions will be analyzed including sensory motor and mid-temporal cortex, cortical gray matter, subcortical gray matter, cerebellum, hippocampus, white matter, and cerebrospinal fluid. Absolute as well as standardized brain volumes (adjusted for PMA at MRI scan: adjusted for total intracranial volume) will be compared in the two groups.

MRI Acquisition Details	T1 weighted 3D SPGR	PD & T2 weighted
**Sequence**	Turbo SPGR	Fast/turbo spin echo ETL/Turbo factor
**TR**	20 ms	10,000 ms
**TE**	9.3 ms	12 ms; 175 ms
**Flip angle**	20°	
**Slice thickness**	1 mm (Gap = 0)	2 mm (Gap = 0)
**Slice orientation**	Axial (parallel to genu & splenium of CC)	Axial (parallel to genu & splenium of CC)
**No. of slices**	Apex of head to bottom of cerebellum (124)	Apex of head to bottom of cerebellum
**FoV**	18 cm	18 cm
**MA**	AP: 256 mm; LR: 256 mm	AP: 256 mm; LR: 192 mm

**NEX**	1	1

APPENDIX IV

Definitions – Qualitative MRI evaluation
Qualitative MRI images will be scored as follows:
Normal: No identifiable lesions or abnormal signal intensity.
Mild: Abnormal focal white matter signal intensity.
Moderate to severe abnormality: Diffuse excessive high signal intensity (DEHSI)[70],[71] and/or diffuse loss of white matter as represented by cystic white matter changes, porencephaly, and/or ventriculomegaly.

Definitions – Cranial US evaluation
Cranial US images will be scored as follows:
Normal**:** No identifiable lesion on imaging.
Mild**:** Isolated germinal matrix or intraventricular hemorrhage without ventricular dilatation (GM/IVH).
Moderate to severe abnormality**:** parenchymal echodensities/echolucencies or ventriculomegaly with or without GM/IVH.[72] This includes grades 3 and 4 IVH.[73]

Neurodevelopmental Outcome Definitions and Determination
Moderate to severe neurosensory deficit at ≥18 months is defined as the presence of one or more of the following diagnoses:
Moderate to severe CP: presence of any 2 of the following 3 abnormalities: 1) delay in motor milestones (PDI ≤ 70 or no walking at 18-22 months), 2) abnormalities observed in the neuromotor exam (tone, deep tendon reflexes, coordination, and movement), and 3) aberrations in primitive reflexes or postural reactions.
Moderate to severe cognitive delay: MDI score < 70 (2 SDs below mean of 100) on the Bayley Scales of Infant Development II.
Bilateral blindness: bilateral blindness with only form or shadow vision or no useful vision.
Bilateral hearing loss: bilateral hearing impairment requiring amplification.

Neurosensory impairment will be assessed at 18-22 months adjusted age according to the NICHD Neonatal Network protocol for all ELBW infants at MHCH.[74] The assessment consists of a developmental evaluation (Bayley Scales of Infant Development II), neurological assessment, (active muscle tone, passive muscle tone, range of motion, reflexes) and functional performance (reflected in the Bayley scale). Trained and certified developmental specialists will perform the Bayley Scales. Physicians will perform neurologic examinations. The exam will include an evaluation for tone (active and passive), strength, reflexes, and posture. In addition, a basic, functional, gross motor skills assessment is performed and scored in the following domains: axis-head and neck, axis-trunk, lower limb function-gait, upper limb function, and hand function. Hearing impairment is defined as any restriction or lack of ability to perform within the normal range and include sensorineural, conductive, or mixed loss. Standard eye examination is also completed to evaluate for esotropia, exotropia, nystagmus, or roving eye movements. Vision impairment is defined as a lack of normal vision including use of corrective lenses or contact lenses, blind with only light or shadow vision or no useful vision. Partly because of the extensive effort used in our clinic to avoid loss to follow-up, the rate of follow-up at 18-22 months adjusted age was 91% in 2002. We will continue to attempt to locate and evaluate any infant lost to follow-up in our usual 18-22 month testing window. With increasing age, the evaluations become more reliable and have greater validity in predicting later impairments. Thus, delays in testing such infants would not invalidate the findings.

APPENDIX V

REFERENCES

[1] Vohr BR, Wright LL, Dusick AM, Mele L, Verter J, Steichen JJ, Simon NP, Wilson DC, Broyles S, Bauer CR, Delaney-Black V, Yolton KA, Fleisher BE, Papile LA, Kaplan MD. Neurodevelopmental and functional outcomes of extremely low birth weight infants in the National Institute of Child Health and Human Development Neonatal Research Network, 1993-1994. Pediatrics 2000;105(6):1216-26.

[2] Schmidt B, Asztalos EV, Roberts RS, Robertson CM, Sauve RS, Whitfield MF. Impact of bronchopulmonary dysplasia, brain injury, and severe retinopathy on the outcome of extremely low-birth-weight infants at 18 months: results from the trial of indomethacin prophylaxis in preterms. JAMA 2003; 289(9):1124-9.

[3] Stevenson DK, Wright LL, Lemons JA, Oh W, Korones SB, Papile LA, Bauer CR, Stoll BJ, Tyson JE, Shankaran S, Fanaroff AA, Donovan EF, Ehrenkranz RA, Verter J. Very low birth weight outcomes of the National Institute of Child Health and Human Development Neonatal Research Network, January 1993 through December 1994. Am J Obstet Gynecol 1998; (6 Pt 1):1632-9.

[4] Vohr BR, Bell EF, Oh W. Infants with bronchopulmonary dysplasia. Growth pattern and neurologic and developmental outcome. Am J Dis Child. 1982;136(5):443-7.

[5] Singer L, Yamashita T, Lilien L, Collin M, Baley J. A longitudinal study of developmental outcome of infants with bronchopulmonary dysplasia and very low birth weight. Pediatrics 1997; 100(6):987-93.

[6] Ellison PH, Farina MA. Progressive central nervous system deterioration: a complication of advanced chronic lung disease of prematurity. Ann Neurol. 1980;8(1):43-6.

[7] Campbell LR, McAlister W, Volpe JJ. Neurologic aspects of bronchopulmonary dysplasia. Clin Pediatr (Phila). 1988;27(1):7-13.

[8] Smith JF, Reynolds EO, Taghizadeh A. Brain maturation and damage in infants dying from chronic pulmonary insufficiency in the postneonatal period. Arch Dis Child. 1974;49(5):359-66.

[9] Kurzner SI, Garg M, Bautista DB, Bader D, Merritt RJ, Warburton D, Keens TG. Growth failure in infants with bronchopulmonary dysplasia: nutrition and elevated resting metabolic expenditure. Pediatrics 1988; 1(3):379-84.

[10] Dammann O, Leviton A, Bartels DB, Dammann CEL. Lung and brain damage in preterm newborns. Are they related? How? Why? Biol Neonate. 2004; 85:305-313.

[11] Bose CL, Laughon MM. Corticosteroids and chronic lung disease: time for another randomized, controlled trial? Pediatrics. 2005; 115:794.

[12] Kennedy KA. Controversies in the use of postnatal steroids. Semin Perinatol 2001;25(6):397-405.

[13] Barrington KJ. The adverse neuro-developmental effects of postnatal steroids in the preterm infant: a systematic review of RCTs. BMC Pediatr 2001; 1(1):1.

[14] Grier DG, Halliday HL. Corticosteroids in the prevention and management of bronchopulmonary dysplasia. Semin Neonatol. 2003; 8(1):83-91.

[15] Brosnan PG. The hypothalamic pituitary axis in the fetus and newborn. Semin Perinatol 2001; 25(6):371-84.

[16] Doyle LW, Halliday HL, Ehrenkranz RA, Davis PG, Sinclair JC. Impact of postnatal systemic corticosteroids on mortality and cerebral palsy in preterm infants: effect modification by risk for chronic lung disease. Pediatrics. 2005; 115:655-61.

[17] Jobe AH. Postnatal corticosteroids for preterm infants--do what we say, not what we do. N Engl J Med. 2004 Mar 25;350(13):1349-51.

[18] Baud O. Is perinatal dexamethasone treatment safe in preterm infants? Dev Med Child Neurol Suppl 2001; 86:23-25.

[19] Baud O, Laudenbach V, Evrard P, Gressens P. Neurotoxic effects of fluorinated glucocorticoid preparations on the developing mouse brain: role of preservatives. Pediatr Res 2001 50(6):706-11.

[20] Baud O, Foix-L'Helias L, Kaminski M, Audibert F, Jarreau PH, Papiernik E, Huon C, Lepercq J, Dehan M, Lacaze-Masmonteil T. Antenatal glucocorticoid treatment and cystic periventricular leukomalacia in very premature infants. N Engl J Med 1999; 341(16):1190-6.

[21] Andre P, Thebaud B, Odievre MH, Razafimahefa H, Zupan V, Dehan M, Lacaze-Masmonteil T. Methyl-prednisolone, an alternative to dexamethasone in very premature infants at risk of chronic lung disease. Intensive Care Med 2000; 26(10):1496-500.

[22] Watterberg KL, Gerdes JS, Gifford KL, Lin HM. Prophylaxis against early adrenal insufficiency to prevent chronic lung disease in premature infants. Pediatrics 1999; 104(6):1258-63.

[23] Watterberg KL, Gerdes JS, Cole CH, Aucott SW, Thilo EH, Mammel MC, Couser RJ, Garland JS, Rozycki HJ, Leach CL, Backstrom C, Shaffer ML. Prophylaxis of early adrenal insufficiency to prevent bronchopulmonary dysplasia: a multicenter trial. Pediatrics. 2004; 114:1649-57.

[24] Stark AR, Carlo WA, Tyson JE, Papile LA, Wright LL, Shankaran S, Donovan EF, Oh W, Bauer CR, Saha S, Poole WK, Stoll BJ. Adverse effects of early dexamethasone in extremely-low-birth-weight infants. National Institute of Child Health and Human Development Neonatal Research Network. N Engl J Med. 2001; 344(2):95-101.

[25] Herman AC, Paxton JB, Carlisle EM, Gaston BM, Gordon PV. Nitric oxide synthase (NOS) abundance is synergistically decreased by the combination of indomethacin and dexamethasone treatment in the submucosa of the neonatal mouse ileum. Pediatr Res 2003; 53(4):441A.

[26] van der Heide-Jalving M, Kamphuis PJ, van der Laan MJ, Bakker JM, Wiegant VM, Heijnen CJ, Veen S, van Bel F. Short- and long-term effects of neonatal glucocorticoid therapy: is hydrocortisone an alternative to dexamethasone? Acta Paediatr. 2003; 92:827-35.

[27] Watterberg KL. Adrenocortical function and dysfunction in the fetus and neonate. Semin Neonatol. 2004; 9:13-21. Review.

[28] Zentay Z, Sharaf M, Qadir M, Drafta D, Davidson D. Mechanism for dexamethasone inhibition of neutrophil migration upon exposure to lipopolysaccharide in vitro: role of neutrophil interleukin-8 release. Pediatr Res 1999; 46(4):406-10.

[29] Skinner AM, Battin M, Solimano A, Daaboul J, Kitson HF. Growth and growth factors in premature infants receiving dexamethasone for bronchopulmonary dysplasia. Am J Perinatol 1997; 14(9):539-46.

[30] Ferrara TB, Couser RJ, Hoekstra RE. Side effects and long-term follow-up of corticosteroid therapy in very low birthweight infants with bronchopulmonary dysplasia. J Perinatol 1990; 10(2):137-42.

[31] Bensky AS, Kothadia JM, Covitz W. Cardiac effects of dexamethasone in very low birth weight infants. Pediatrics 1996; 97(6 Pt 1):818-21.

[32] Peng CT, Lin HC, Lin YJ, Tsai CH, Yeh TF. Early dexamethasone therapy and blood cell count in preterm infants. Pediatrics 1999; 104(3 Pt 1):476-81.

[33] Parimi PS, Birnkrant DJ, Rao LV, Diaz G, Moore JJ. Effect of dexamethasone on lymphocyte subpopulations in premature infants with bronchopulmonary dysplasia. J Perinatol 1999; 19(5):347-51.

[34] Murphy BP, Inder TE, Huppi PS, Warfield S, Zientara GP, Kikinis R, Jolesz FA, Volpe JJ. Impaired cerebral cortical gray matter growth after treatment with dexamethasone for neonatal chronic lung disease. Pediatrics 2001; 107(2):217-21.

[35] Huppi PS, Warfield S, Kikinis R, Barnes PD, Zientara GP, Jolesz FA, Tsuji MK, Volpe JJ. Quantitative magnetic resonance imaging of brain development in premature and mature newborns. Ann Neurol 1998; 43(2):224-35.

[36] Parikh NA, Lasky RE, Tyson TE, Moya FR. Kennedy KA. Effects of Postnatal Dexamethasone on Cerebral Tissue Volumes in Extremely Low Birth Weight Infants. Pediatr Res. 57(4):1505A, 2005.

[37] Durand M, Mendoza ME, Tantivit P, Kugelman A, McEvoy C. A randomized trial of moderately early low-dose dexamethasone therapy in very low birth weight infants: dynamic pulmonary mechanics, oxygenation, and ventilation. Pediatrics. 2002; 109(2):262-8.

[38] Weld C, Sardesai S, Siassi B, Seri I, Ramanathan R. Very low doses of dexamethasone for the treatment of respiratory failure in very low birth weight infants with evolving bronchopulmonary dysplasia: Neurodevelopmental outcome at 18 to 24 months of age. Pediatr Res. 2003; 3(4):513A.

[39] Halliday HL, Ehrenkranz RA, Doyle LW. Early postnatal (<96 hours) corticosteroids for preventing chronic lung disease in preterm infants. [Systematic Review] Cochrane Database of Systematic Reviews. 2003;(1):CD001146.

[40] Halliday HL, Ehrenkranz RA, Doyle LW. Moderately early (7-14 days) postnatal corticosteroids for preventing chronic lung disease in preterm infants. [Systematic Review] Cochrane Database of Systematic Reviews. 2003;(1):CD001144.

[41] Halliday HL, Ehrenkranz RA, Doyle LW. Delayed (>3 weeks) postnatal corticosteroids for chronic lung disease in preterm infants. [Systematic Review] Cochrane Database of Systematic Reviews. 2003;(1):CD001145.

[42] Grier DG, Halliday HL. Management of bronchopulmonary dysplasia in infants: guidelines for corticosteroid use. Drugs. 2005; 65:15-29.

[43] Yoder BA, Anwar MU, Clark RH. Early prediction of neonatal chronic lung disease: a comparison of three scoring methods. Pediatr Pulmonol. 1999; 27:388-94.

[44] Papile LA, Tyson JE, Stoll BJ, Wright LL, Donovan EF, Bauer CR, Krause-Steinrauf H, Verter J, Korones SB, Lemons JA, Fanaroff AA, Stevenson DK. A multicenter trial of two dexamethasone regimens in ventilator-dependent premature infants. N Engl J Med 1998; 338(16):1112-8.

[45] American Academy of Pediatrics and Canadian Paediatric Society. Postnatal corticosteroids to treat or prevent chronic lung disease in preterm infants. Pediatrics 2002; 109(2) 330-8.

[46] Soll, RF. Corticosteroids for the treatment and prevention of chronic lung disease. Acta Paediatr 2003; 92:886-889.

[47] Thebaud B, Lacaze-Masmonteil T, Watterberg K. Postnatal glucocorticoids in very preterm infants: "the good, the bad, and the ugly"? Pediatrics 2001; 107(2):413-5.

[48] Perlman J. Concern about Fetus and Newborn Committee statement on corticosteroid use. Pediatrics 2002; 110(5):1034.

[49] Taylor C, Shah PS, Dunn MS. Meta-analysis of postnatal steroid use challenged. Pediatrics. 2002;109(4):716-7

[50] Jacobs HC, Chapman RL, Gross I. Premature conclusions on postnatal steroid effects. Pediatrics 2002; 110(1 Pt 1):200-1.

[51] Peltoniemi O, Kari MA, Heinonen K, Saarela T, Nikolajev K, Andersson S, Voutilainen R, Hallman M. Pretreatment cortisol values may predict responses to hydrocortisone administration for the prevention of bronchopulmonary dysplasia in high-risk infants. J Pediatr. 2005; 146(5):632-7.

[52] Caeymaex L, Scheid A, Gatel P, Coulomb A, Zupan-Simunek V, Lacaze-Masmonteil T. Is prophylactic treatment with low-dose hydrocortisone in ELBW infants associated with gastro-intestinal perforation? Pediatr Res. 2005; 57(4): 3383A.

[53] Herman AC, Paxton JB, Carlisle EM, Gaston BM, Gordon PV. Nitric oxide synthase (NOS) abundance is synergistically decreased by the combination of indomethacin and dexamethasone treatment in the submucosa of the neonatal mouse ileum. Pediatr Res 2003; 53(4):441A.

[54] Groneck P, Speer CP. Inflammatory mediators and bronchopulmonary dysplasia. Arch Dis Child Fetal Neonatal Ed. 1995; 73(1):F1-3.

[55] Jobe AH. The New BPD: an arrest of lung development. Pediatr Res 1999; 46:641-643.

[56] Lodygensky GA, Rademaker K, Zimine S, Gex-Fabry M, Lieftink AF, Lazeyras F, Groenendaal F, de Vries LS, Huppi PS. Structural and functional brain development after hydrocortisone treatment for neonatal chronic lung disease. Pediatrics. 2005; 116(1):1-7.

[57] Papile LA, Krause-Steinrauf H, Wright LL, Stoll BJ, Donovan EF, Bauer CR, Tyson JE, Verter J For the NICHD Neonatal Network. Identification of very low birthweight infants (VLBW) at risk for chronic lung disease (CLD). Pediatr Res 1994; 35:246A.

[58] Halliday HL. Postnatal steroids: a dilemma for the neonatologist. Acta Paediatr 2001; 90(2):116-8.

[59] The STOP-ROP Multicenter Study Group. Supplemental therapeutic oxygen for prethreshold retinopathy of prematurity (STOP-ROP), a randomized, controlled trial I: primary outcomes. Pediatrics 2000; 105(2):295-310.

[60] Tin W, Milligan DW, Pennefather P, Hey E. Pulse oximetry, severe retinopathy, and outcome at one year in babies of less than 28 weeks gestation. Arch Dis Child Fetal Neonatal Ed 2001; 84(2):F106-10.

[61] Mariani G, Cifuentes J, Carlo W. Randomized trial of permissive hypercapnea in preterm infants. Pediatrics 1999; 104:1082-8.

[62] Lasky RE, Luck ML, Parikh NA, Laughlin NK. The Effects of Early Lead Exposure on the Brains of Adult Rhesus Monkeys: A Volumetric MRI Study. Toxicol Sci. 2005; 85(2):963-975.

[63] Ajayi-Obe M, Saeed N, Cowan FM, Rutherford MA, Edwards AD. Reduced development of cerebral cortex in extremely preterm infants. Lancet 2000; 356(9236):1162-3.

[64] Jobe AH, Bancalari E. Bronchopulmonary dysplasia. Am J Respir Crit Care Med 2001; 163(7):1723-9.

[65] Bancalari E, Claure N, Sosenko IR. Bronchopulmonary dysplasia: changes in pathogenesis, epidemiology and definition. Semin Neonatol 2003; 8(1):63-71.

[66] Donovan EF, Tyson JE, Ehrenkranz RA, Verter J, Wright LL, Korones SB, Bauer CR, Shankaran S, Stoll BJ, Fanaroff AA, Oh W, Lemons JA, Stevenson DK, Papile LA. Inaccuracy

of Ballard scores before 28 weeks' gestation. National Institute of Child Health and Human Development Neonatal Research Network. J Pediatr. 1999;135(2 Pt 1):147-52.

[67] Jones RA, Palasis S, Grattan-Smith JD. MRI of the neonatal brain: optimization of spin-echo parameters. AJR Am J Roentgenol. 2004;182:367-72.

[68] Counsell SJ, Kennea NL, Herlihy AH, Allsop JM, Harrison MC, Cowan FM, Hajnal JV, Edwards B, Edwards AD, Rutherford MA. T2 relaxation values in the developing preterm brain. AJNR Am J Neuroradiol. 2003; 24:1654-60.

[69] AnalyzeDirect: Comprehensive Visualization for Biomedical Images. [Online] http://www.analyzedirect. com.

[70] Maalouf EF, Duggan PJ, Rutherford MA, Counsell SJ, Fletcher AM, Battin M, Cowan F, Edwards AD. Magnetic resonance imaging of the brain in a cohort of extremely preterm infants. J Pediatr. 1999;135:351-7.

[71] Counsell SJ, Allsop JM, Harrison MC, Larkman DJ, Kennea NL, Kapellou O, Cowan FM, Hajnal JV, Edwards AD, Rutherford MA.Diffusion-weighted imaging of the brain in preterm infants with focal and diffuse white matter abnormality. Pediatrics. 2003l; 112:1-7.

[72] Pinto-Martin JA, Riolo S, Cnaan A, Holzman C, Susser MW, Paneth N. Cranial ultrasound prediction of disabling and nondisabling cerebral palsy at age two in a low birth weight population. Pediatrics 1995; 95(2):249-54.

[73] Papile LA, Burstein J, Burstein R, Koffler H. Incidence and evolution of subependymal and intraventricular hemorrhage: a study of infants with birth weights less than 1,500 gm. J Pediatr. 1978; 92:529-34.

[74] Vohr BR, Wright LL, Dusick AM, Mele L, Verter J, Steichen JJ, Simon NP, Wilson DC, Broyles S, Bauer CR, Delaney-Black V, Yolton KA, Fleisher BE, Papile LA, Kaplan MD. Neurodevelopmental and functional outcomes of extremely low birth weight infants in the National Institute of Child Health and Human Development Neonatal Research Network, 1993-1994. Pediatrics. 2000 Jun;105(6):1216-26.
